# Spermatozoa Develop Molecular Machinery to Recover From Acute Stress

**DOI:** 10.3389/fendo.2022.896193

**Published:** 2022-07-14

**Authors:** Isidora M. Starovlah, Sava M. Radovic Pletikosic, Tamara M. Tomanic, Marija LJ. Medar, Tatjana S. Kostic, Silvana A. Andric

**Affiliations:** Laboratory for Reproductive Endocrinology and Signaling, Laboratory for Chronobiology and Aging, Center for Reproductive Endocrinology and Signaling, Department of Biology and Ecology, Faculty of Sciences, University of Novi Sad, Novi Sad, Serbia

**Keywords:** acute psychological stress, stress recovery, mitochondrial dynamics and functionality markers, cAMP signaling markers, MAPK signaling markers, spermatozoa number and functionality

## Abstract

This study was designed to search for the possible mechanism(s) of male (in/sub)fertility by following the molecular response of spermatozoa on acute psychological stress (the most common stress in human society) and on a 20-h time-dependent recovery period. To mimic *in vivo* acute stress, the rats were exposed to immobilization once every 3 h. The recovery periods were as follows: 0 (immediately after stress and 3 h after the light is on—ZT3), 8 (ZT11), 14 (ZT17), and 20 (ZT23) h after stress. Results showed that acute stress provoked effects evident 20 h after the end of the stress period. Numbers of spermatozoa declined at ZT17 and ZT23, while functionality decreased at ZT3 and ZT11, but recovered at ZT17 and ZT23. Transcriptional profiles of 91% (20/22) of tracked mitochondrial dynamics and functionality markers and 91% (20/22) of signaling molecules regulating both mitochondrial dynamics and spermatozoa number/functionality were disturbed after acute stress and during the recovery period. Most of the changes presented as increased transcription or protein expression at ZT23. The results of the principal component analysis (PCA) showed the clear separation of acute stress recovery effects during active/dark and inactive/light phases. The physiological relevance of these results is the recovered positive-acrosome-reaction, suggesting that molecular events are an adaptive mechanism, regulated by acute stress response signaling. The results of the PCA confirmed the separation of the effects of acute stress recovery on gene expression related to mitochondrial dynamics, cAMP, and MAPK signaling. The transcriptional patterns were different during the active and inactive phases. Most of the transcripts were highly expressed during the active phase, which is expected given that stress occurred at the beginning of the inactive phase. To the best of our knowledge, our results provide a completely new view and the first presentation of the markers of mitochondrial dynamics network in spermatozoa and their correlation with signaling molecules regulating both mitochondrial dynamics and spermatozoa number and functionality during recovery from acute stress. Moreover, the interactions between the proteins important for spermatozoa homeostasis and functionality (MFN2 and PRKA catalytic subunit, MFN2 and p38MAPK) are shown for the first time. Since the existing literature suggests the importance of semen quality and male fertility not only as the fundamental marker of reproductive health but also as the fundamental biomarkers of overall health and harbingers for the development of comorbidity and mortality, we anticipate our result to be a starting point for more investigations considering the mitochondrial dynamics markers or their transcriptional profiles as possible predictors of (in/sub)fertility.

## Introduction

Stress is an important adaptive response of an organism that enables survival and maintains homeostasis (1). However, if it is repeated or persistent/chronic, it can cause diseases (2–6). Many epidemiological studies showed that DNA damage during stress response is regulated through adrenergic signaling (7). It is clear that different types of stress and stressful life events have been linked to reduced adult male reproductive function (8–11). Numerous studies reported connection between male (sub/in)fertility and stressful life (8, 12–14). However, mechanisms causing the (sub/in)fertility are not described yet.

Mitochondria are a very important linking point between stress response and spermatozoa functionality since these organelles are able to produce enormous levels of energy required for both processes (2, 3, 6, 13). Moreover, signaling pathways activated by stress hormone receptors are important for homeostasis of mitochondrial network and spermatozoa functionality (10, 11, 15). The mtDNA is required for male fertility (16) and could be a diagnostic marker for sperm quality in men (17). The disturbed mtDNA was observed in oligo-asthenozoospermic patients (18) and in asthenoteratozoospermia-induced male infertility (19). Since the mitochondrial morphology changes during spermatogenesis (20), the disturbed ultrastructure of mitochondria can explain some of the unexplained cases of asthenozoospermia (21). Moreover, the mitochondrial membrane potential is also important for spermatozoa functionality (22–25). The reduced mtDNA content in human sperm (26) and the expression of TFAM gene correlate with abnormal spermatozoa forms (27, 28). Furthermore, human sperm motility and viability are regulated by mitophagy (29) as well as UCP2 (30) and the MFN2 expression levels (31). Thus, the mitochondria are a crucial organelle for spermatozoa wellbeing and fertility (13).

The homeostasis of the mitochondrial network is regulated by intriguing processes of mitochondrial dynamics including mitochondrial biogenesis, mitofusion, mitofission, and mitophagy (32–35). All processes involve a complex and multistep molecular event required for renewal, adaptation, or expansion of the mitochondrial network (26, 36–38). The main molecular markers of mitochondrial dynamics are not only the main markers of mitochondrial biogenesis (PGC1α, PGC1β, NRF1, NRF2, and TFAM), mitofusion (MFN1, MFN2, and OPA1), mitofission (DRP1 and FIS1), and mitophagy (PINK1 and PARKIN), but also important markers of the respiratory chain function (32–35, 37, 38). In addition, maintaining homeostasis of the mitochondrial network requires intriguing and complex network of signaling pathways (33, 36, 38), which are able to convey a wide variety of different environmental signals: stress (39, 40), temperature (41), energy deprivation (38), availability of nutrients (38), and growth factors (42).

It is important to point out that all signaling pathways regulating mitochondrial dynamics are required for spermatozoa homeostasis (43). Similar signaling pathways are involved in regulation of the function of sperm flagellum (44). Additional complications related to understanding the regulation of spermatozoa functionality are findings that show that murine germ cells highly express genes involved in steroidogenesis and other cell functions, such as genes involved in fatty acid metabolism or synthesis. This supports the possibility of an additional level of regulation of spermatogenesis (45, 46).

**Figure 1:**
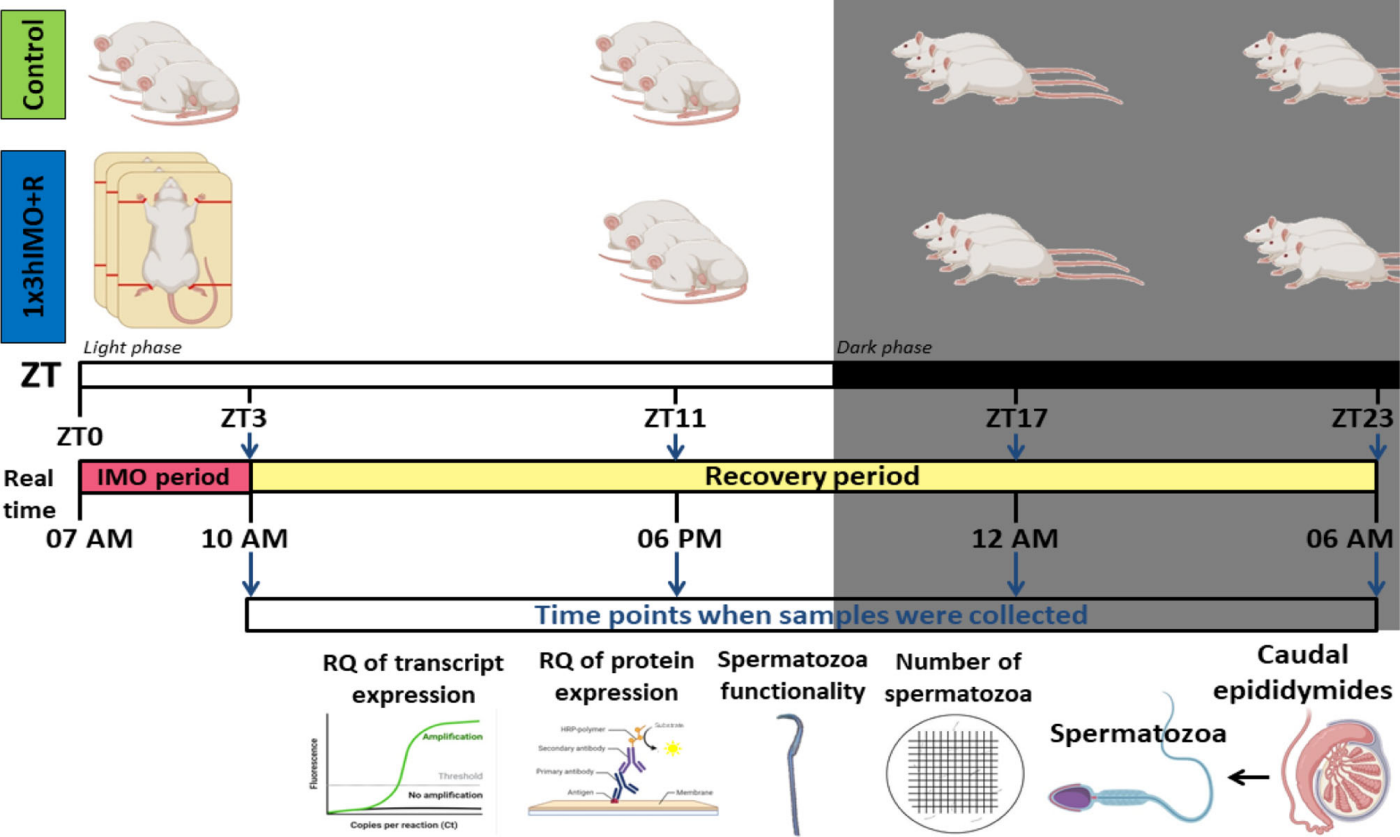
Experimental design of immobilization stress with recovery period used to assess spermatozoa number and functionality (% acrosome reaction) as well as mitochondrial dynamics markers and related signaling molecule expression profiles of transcripts and proteins.

**Figure 2:**
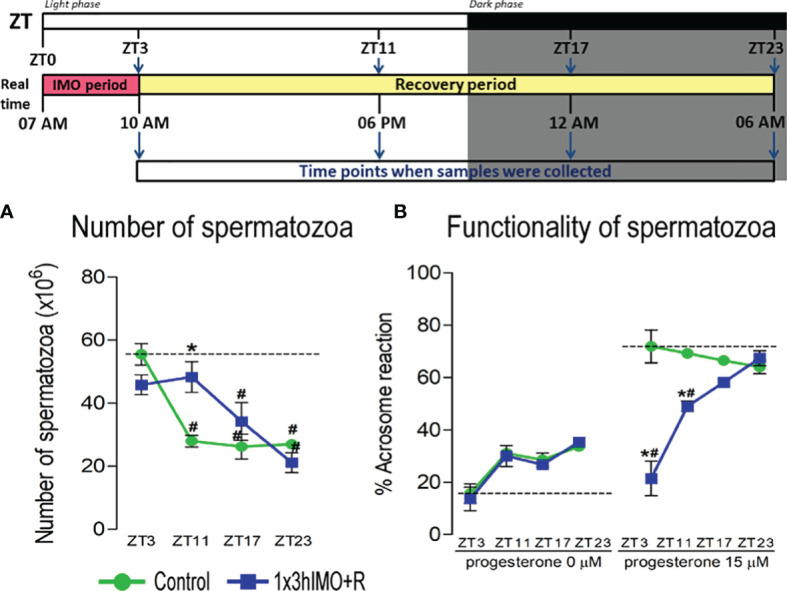
The acute psychophysical stress by immobilization (IMO) decreases functionality and number of spermatozoa in different time points after the IMO stress. Number of spermatozoa **(A)** isolated from caudal epididymides of unstressed rats (control) and rats subjected to acute immobilization stress once for 3 h (1x3hIMO) with recovery periods of 0, 8, 14 and 20 h. **(B)** The functionality of spermatozoa (% of acrosome reacted spermatozoa) isolated from control and acutely (1x3hIMO) stressed rats. Capacitated spermatozoa were stimulated with progesterone (PROG 15 µM) in parallel with spermatozoa not treated with progesterone (PROG 0 µM). Blue staining in the acrosome region of the head indicated intact acrosome, whereas spermatozoa without blue staining in the acrosome region were considered to be acrosome reacted. Data are presented as green dots connected with a green line for the control group, and blue squares connected with a blue line for the 1x3hIMO group, and are mean ± SEM values of two independent *in vivo* experiments. Statistical significance was set at *p* < 0.05: * vs. the control group of the same time point, # vs. the control group of ZT3 time point.

**Figure 3:**
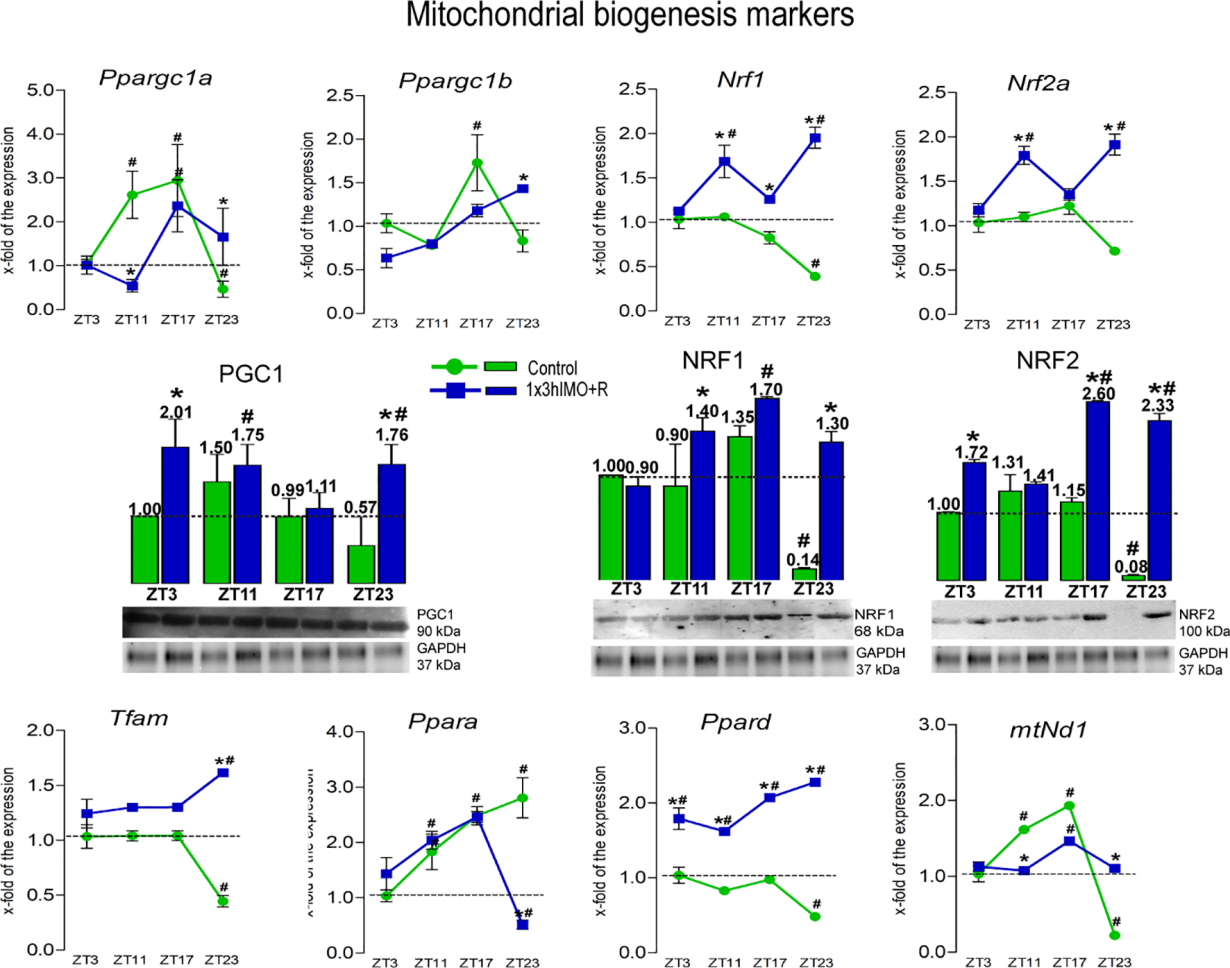
Transcription of mitochondrial biogenesis markers is significantly changed in spermatozoa of acutely stressed adult rats in a time-dependent manner. Isolated RNA and proteins from spermatozoa of undisturbed and stressed rats were used for the analysis of the transcriptional profile and protein expression profile of markers of mitochondrial biogenesis. The representative blots are shown as panels. Data from scanning densitometry were normalized on GAPDH (internal control). Values are shown as bars above the photos of blots. Data are presented as green dots connected with a green line for the control group, and blue squares connected with a blue line for the 1x3hIMO group, and are mean ± SEM values of two independent *in vivo* experiments. Statistical significance was set at *p* < 0.05: * vs. the control group of the same time point, ^#^ vs. the control group of ZT3 time point.

**Figure 4:**
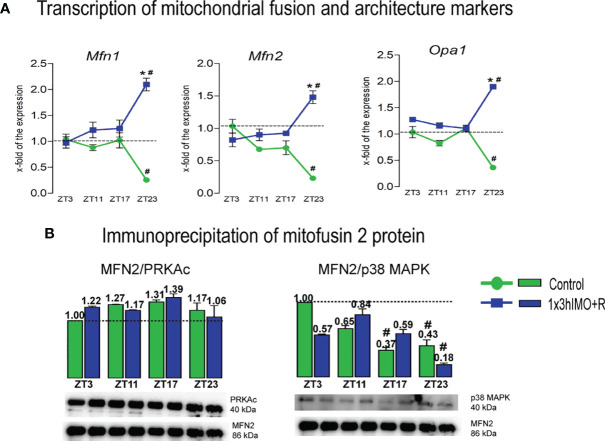
Transcription of mitochondrial fusion and architecture markers, as well as interactions of mitofusin 2 protein and PRKAc and p38 MAPK proteins are significantly changed in spermatozoa of acutely stressed adult rats in a time-dependent manner. Isolated RNA from spermatozoa of undisturbed and stressed rats was used for the analysis of the transcriptional profile of markers of mitochondrial fusion and architecture **(A)**. Isolated proteins from spermatozoa of undisturbed and stressed rats were used for immunoprecipitation analysis with MFN2 antibody, followed by Western blot for PRKAc and p38 MAPK **(B)**. The representative blots are shown as panels. Data from scanning densitometry were normalized on MFN2 (internal control). Values are shown as bars above the photos of blots. Data are presented as green dots connected with a green line or green bars for the control group, and blue squares connected with a blue line or blue bars for the 1x3hIMO group, and are mean ± SEM values of two independent *in vivo* experiments. Statistical significance was set at *p* < 0.05: * vs. the control group of the same time point, # vs. the control group of ZT3 time point.

**Figure 5:**
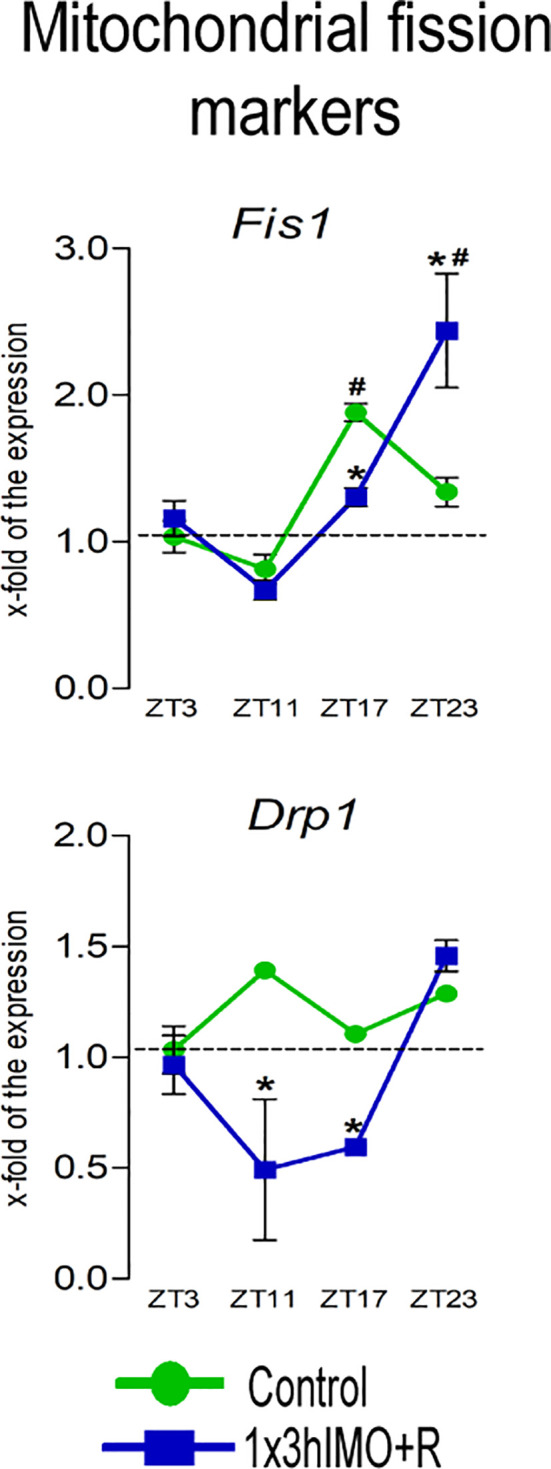
Transcription of mitochondrial fission markers is significantly changed in spermatozoa of acutely stressed adult rats in a time-dependent manner. Isolated RNA from spermatozoa of undisturbed and stressed rats was used for the analysis of the transcriptional profile of markers of mitochondrial fission. Data are presented as green dots connected a with green line for the control group, and blue squares connected with a blue line for the 1x3hIMO group, and are mean ± SEM values of two independent *in vivo* experiments. Statistical significance was set at *p* < 0.05: * vs. the control group of the same time point, # vs. the control group of ZT3 time point.

**Figure 6:**
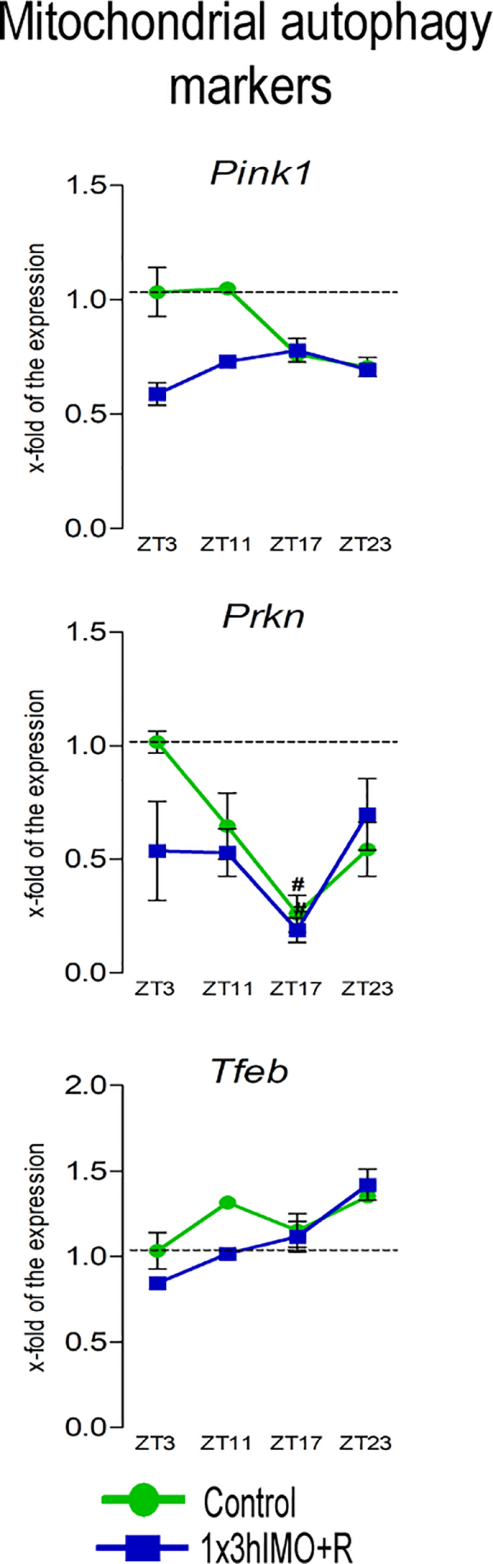
Transcription of mitochondrial autophagy markers is significantly changed in spermatozoa of acutely stressed adult rats in a time-dependent manner. Isolated RNA from spermatozoa of undisturbed and stressed rats was used for the analysis of the transcriptional profile of markers of mitochondrial autophagy. Data are presented as green dots connected with a green line for the control group, and blue squares connected with a blue line for the 1x3hIMO group, and are mean ± SEM values of two independent *in vivo* experiments. Statistical significance was set at *p* < 0.05: * vs. the control group of the same time point, ^#^ vs. the control group of ZT3 time point.

**Figure 7:**
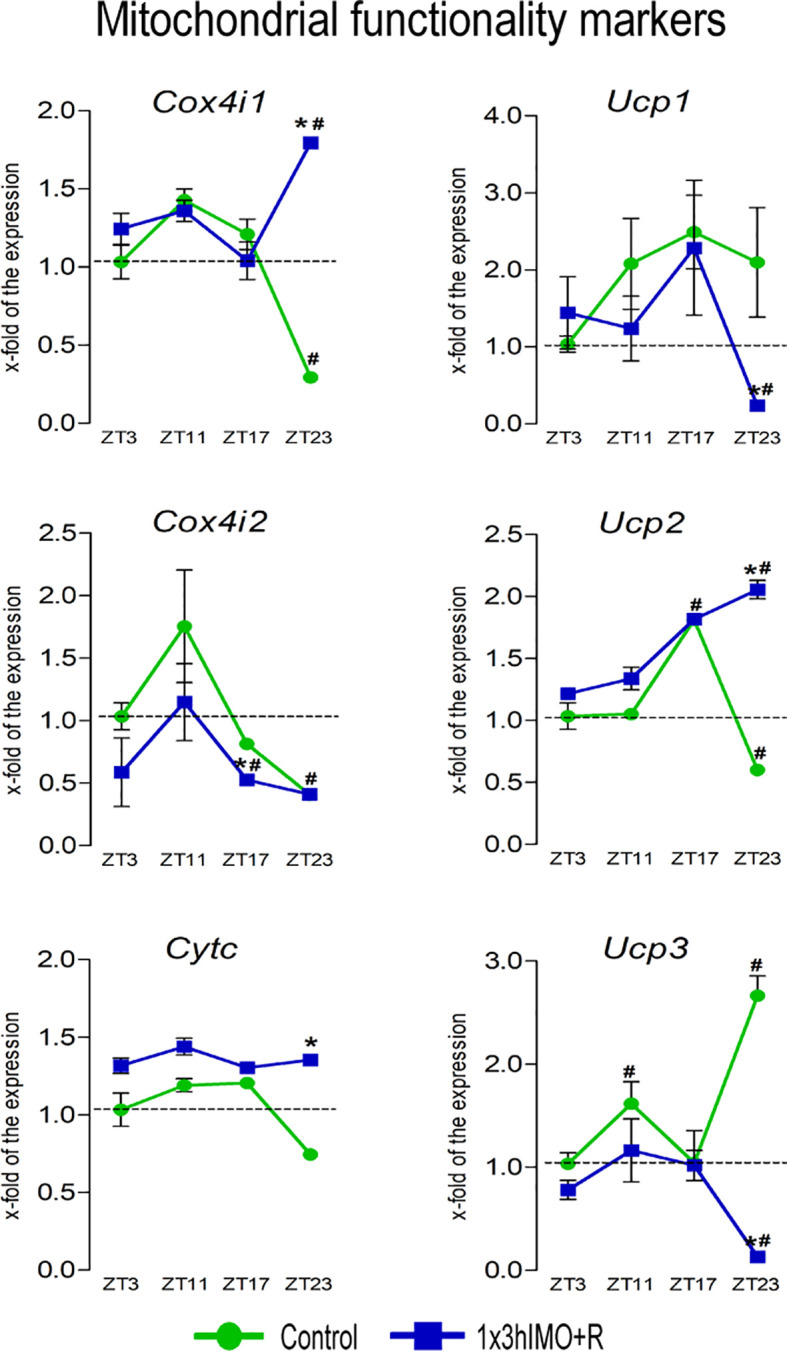
Transcription of mitochondrial functionality markers is significantly changed in spermatozoa of acutely stressed adult rats in a time-dependent manner. Isolated RNA from spermatozoa of undisturbed and stressed rats was used for the analysis of the transcriptional profile of markers of mitochondrial functionality. Data are presented as green dots connected with a green line for the control group, and blue squares connected with a blue line for the 1x3hIMO group, and are mean ± SEM values of two independent *in vivo* experiments. Statistical significance was set at *p* < 0.05: * vs. the control group of the same time point, ^#^ vs. the control group of ZT3 time point.

**Figure 8:**
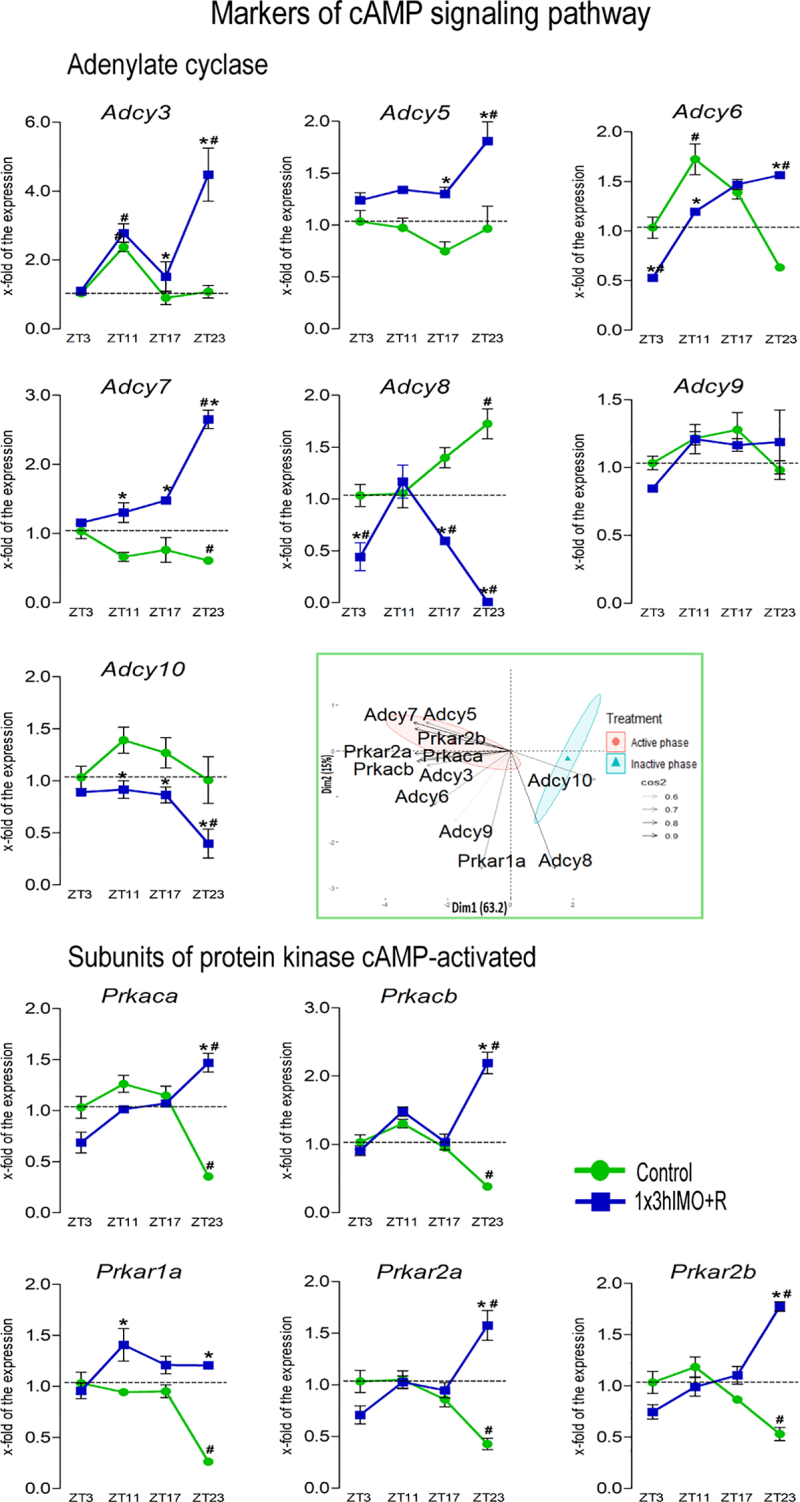
Transcription of markers of cAMP signaling regulating mitochondrial dynamics and functionality as well as spermatozoa number and functionality is changed in spermatozoa of acutely stressed adult rats in a time-dependent manner. Isolated RNA from spermatozoa of undisturbed and stressed rats was used for the analysis of the transcriptional profile of markers of the cAMP signaling pathway. PCA of markers of the cAMP signaling pathway on active/inactive phase; Dim1 and Dim2 represent the first two PCs and % of the retained variation. Cos2 estimates the qualitative representation of variables (**Supplementary Table S2**). Data are presented as green dots connected with a green line for the control group, and blue squares connected with a blue line for the 1x3hIMO group, and are mean ± SEM values of two independent *in vivo* experiments. Statistical significance was set at *p* < 0.05: * vs. the control group of the same time point, # vs. the control group of ZT3 time point.

**Figure 9:**
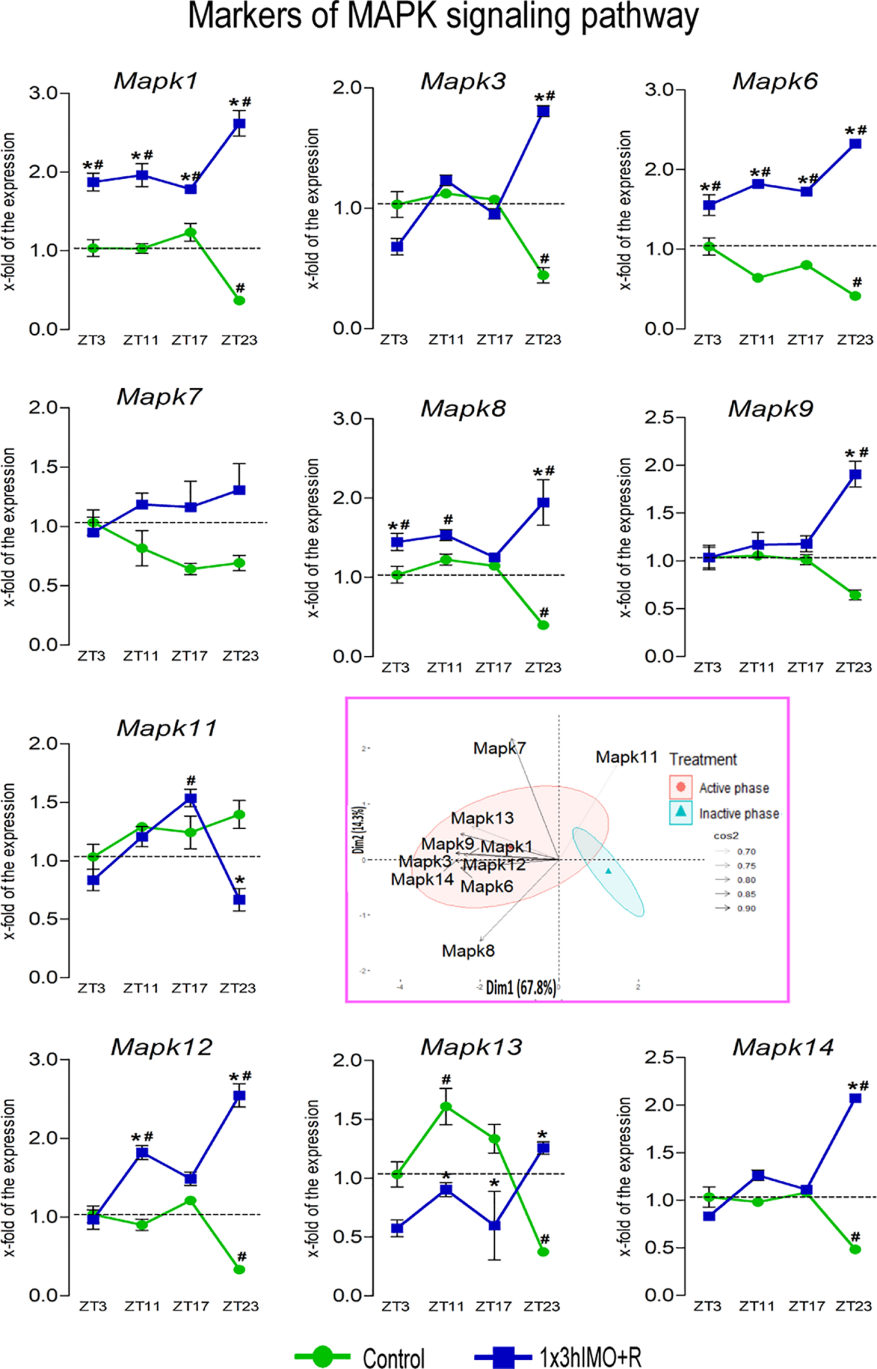
Transcription of markers of MAPK signaling regulating mitochondrial dynamics and functionality as well as spermatozoa number and functionality is changed in spermatozoa of acutely stressed adult rats in a time-dependent manner. Isolated RNA from spermatozoa of undisturbed and stressed rats was used for the analysis of the transcriptional profile of markers of the MAPK pathway. PCA of markers of the MAPK signaling pathway on active/inactive phase; Dim1 and Dim2 represent the first two PCs and % of the retained variation. Cos2 estimates the qualitative representation of variables (**Supplementary Table S3**). Data are presented as green dots connected with a green line for the control group, and blue squares connected with a blue line for the 1x3hIMO group, and are mean ± SEM values of two independent *in vivo* experiments. Statistical significance was set at *p* < 0.05: * vs. the control group of the same time point, # vs. the control group of ZT3 time point.

**Figure 10:**
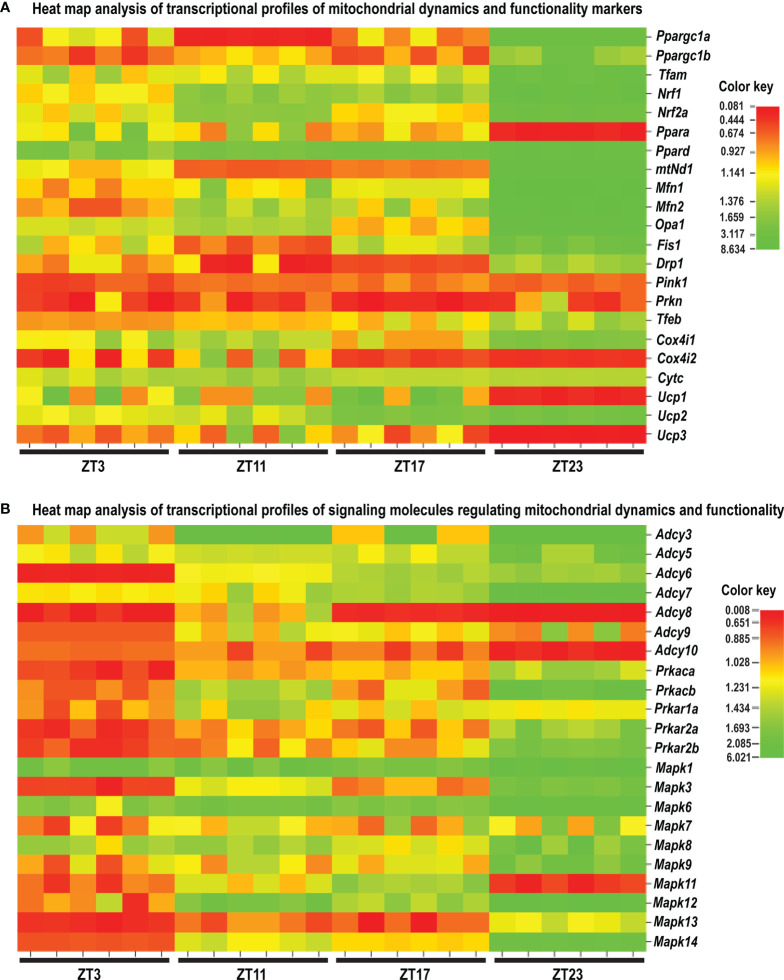
Heat map analysis of the transcriptional profile of the mitochondrial dynamic and functionality markers **(A)** and the signaling molecules regulating mitochondrial dynamics and functionality **(B)** in spermatozoa of acute stressed adult rats. Heat map analysis showing different patterns of transcription at different time points in spermatozoa after the acute immobilization stress. The relative fold change in gene expression for the aforementioned genes was compared in different time points (ZT3, ZT11, ZT17, and ZT23). Color from red to green indicates low to high expression.

**Figure 11:**
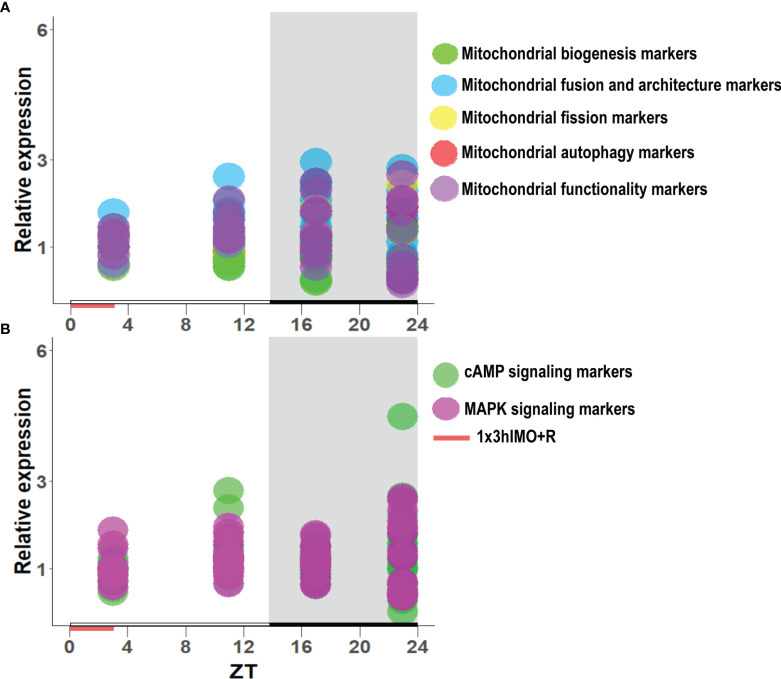
The transcription pattern in spermatozoa of acutely stressed adult rats with different recovery periods (ZT3, ZT11, ZT17, and ZT23). Data shown represent the transcriptional pattern of the genes for mitochondrial dynamics/functionality markers **(A)** as well as cAMP and MAPK signaling pathway-related molecules **(B)**. Points represent a deviation in the transcription of a particular gene at different ZT time points.

**Figure 12:**
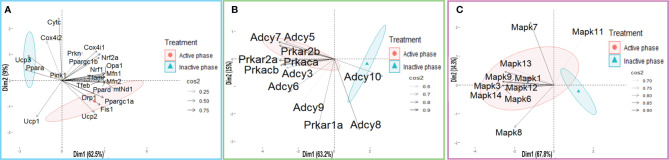
PCA of mitochondrial dynamics **(A)**, cAMP signaling pathway **(B)**, and MAPK signaling pathway **(C)** gene expression on active/inactive phase; Dim1 and Dim2 represent the first two PCs and % of the retained variation. Cos2 estimates the qualitative representation of variables (**Supplementary Tables S1–S3**).

**Figure 13:**
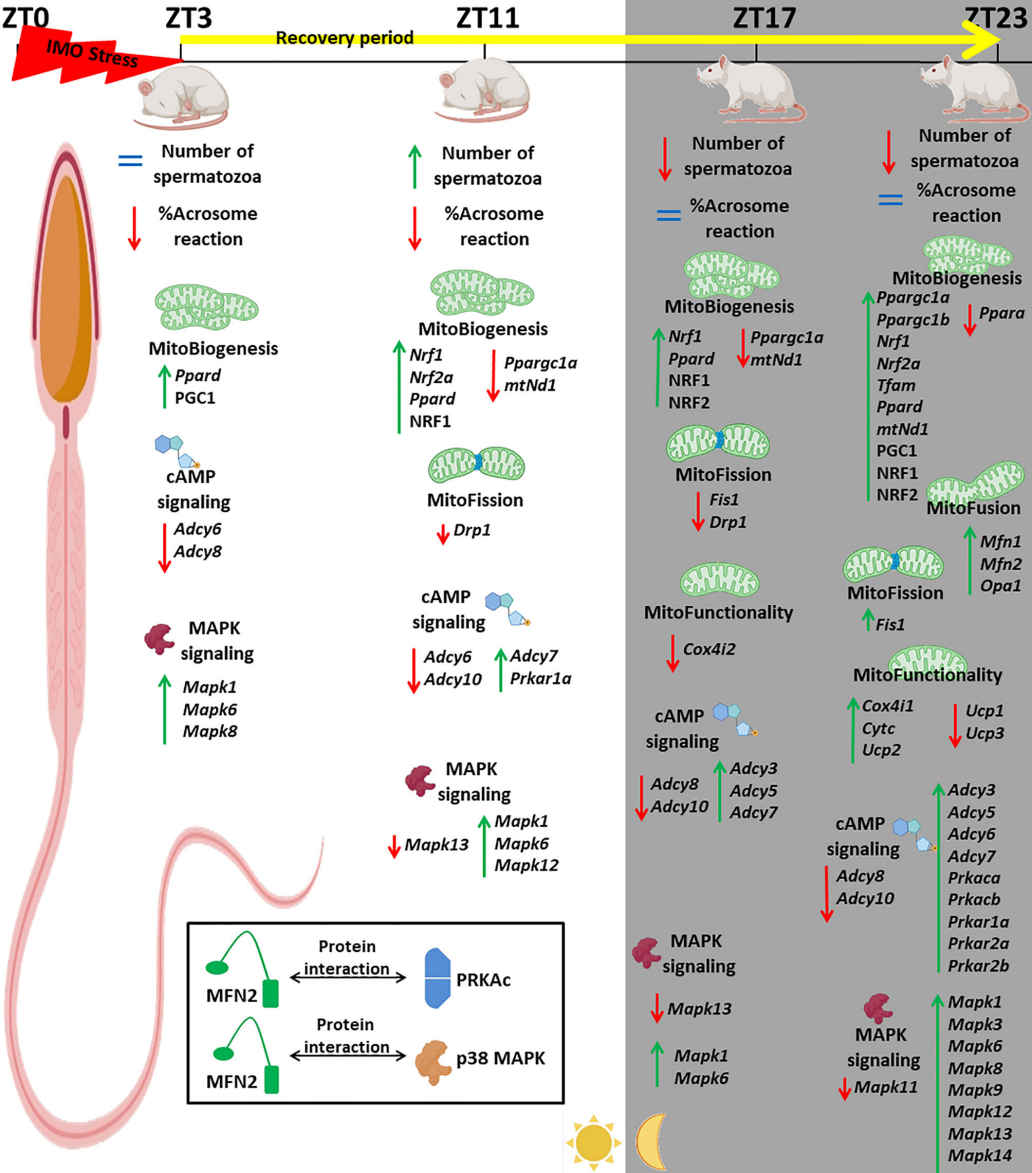
Acute stress, the most common stress in human society, significantly changes 91% of followed mitochondrial dynamics and functionality markers as well as 91% of signaling molecules regulating spermatozoa homeostasis and mitochondrial dynamics/functionality. The most prominent changes were observed 20 h after the end of the stress. The physiological significances are the recovery of spermatozoa number and functionality (positive acrosome reaction). Furthermore, the interactions between the proteins important for spermatozoa homeostasis and functionality (MFN2 and PRKA catalytic subunit, MFN2, and p38MAPK) are shown for the first time.

In search for mechanisms activated by and during stress, we explored molecular events in spermatozoa at four time points in a 20-h time-dependent recovery period after acute stress (once for the duration of 3 h, 7 a.m. to 10 a.m.). Acute stress was chosen since it is the most common stress in human society. Recovery was followed at a different time points during the day (light/inactive and dark/active phase): immediately after acute stress (ZT3) as well as 8 (ZT11), 14 (ZT17), and 20 (ZT23) h after acute stress. Number and functionality of spermatozoa, as well as the transcriptional profiles of 22 mitochondrial dynamics and function markers and 22 related signaling molecules were followed (**Figures 1**–**13**). Two rationales were prevalent in the decision to follow spermatozoal functionality by acrosome reaction. First, acrosome reaction is the event in the timeline that is closer to fertilization than motility or other parameters. Second, working with human samples (640 samples were collected over the last 18 months) from men attending the national IVF program, we came to learn that there are significant numbers of normozoospermic samples with good motility and other parameters of spermiogram, but with negative acrosome reaction, suggesting the possible reason for entering the IVF program.

## Materials and Methods

All experiments were carried out in the Laboratory for Reproductive Endocrinology and Signaling and Laboratory for Chronobiology and Aging, Faculty of Sciences at University of Novi Sad (wwwold.dbe.pmf.uns.ac.rs/en/nauka-eng/lares). Methods used in this study were carried out following relevant guidelines and regulations and were reported previously [for all references, please see (10, 11, 47)]. Key resource tables and tables containing primers and antibody data are provided in the **Supplementary Material**.

### Statement of the Institutional Review Board

The manuscript is approved by the Committee of the Faculty of Sciences, University of Novi Sad, Novi Sad, Serbia.

The authors complied with ARRIVE guidelines and all experiments were in adherence to the ARRIVE guidelines. All experimental protocols were approved (statement no. 01-201/3) by the local Ethical Committee on Animal Care and Use of the University of Novi Sad operating under the rules of the National Council for Animal Welfare and the National Law for Animal Welfare (copyright March 2009), following the NRC publication *Guide for the Care and Use of Laboratory Animals* and the NIH *Guide for the Care and Use of Laboratory Animals*.

### Animals and Experimental Model of Acute Stress With a Recovery Period

Adult, 3-month-old, male Wistar rats were used in all experiments. Animals were bred and raised in the accredited Animal Facility of the Faculty of Sciences, University of Novi Sad, Serbia, in controlled environmental conditions [22 ± 2°C; 14-h light and 10-h dark cycle, lights on at 07:00 a.m. (ZT0)] with food and water *ad libitum*. The experimental model of psychophysical stress by immobilization (IMO) was performed by the method previously described (10, 11, 47, 48). To analyze the effects of acute stress with the recovery period (48), animals were subjected to immobilization stress (IMO) for 3 h, once, from ZT0 to ZT3 (1x3hIMO), and allowed to recover (1x3hIMO+R) for 0, 8, 14, and 20 h after the IMO (ZT3, ZT11, ZT17, and ZT23; ZT0 is a time when the light is turned on) (**Figure 1**). The experimental model of psychophysical stress by immobilization was performed by the method previously described (9–11). In short, rats were bound in a supine position to a wooden board by fixing the rats’ limbs using thread, while the head motion was not limited. Unstressed, freely moving rats were present as a control group (Control) in each experiment. All activities during the dark phase were performed under the red light. At the end of the experimental period, control and stressed animals were quickly decapitated without anesthesia and trunk blood was collected. In each experiment, both control and stressed animals were randomly divided into four time point groups, with a total of 4 animals in the control group and 6 animals in the 1x3hIMO+R group per time point. The sample size was checked by Power Analysis using the G Power software (http://core.ecu.edu/psyc/wuenschk/Power.htm) according to previously published results. The experiment was repeated two times.

### Spermatozoa Isolation and Their Functionality Assessment (Capacitation and Acrosome Reaction)

Isolation of caudal epididymides spermatozoa was carried out following the WHO laboratory manual (https://www.who.int/publications/i/item/9789240030787) with modifications for rat spermatozoa isolation. In short, caudal epididymides were quickly isolated, the surrounding adipose tissue was removed, and epididymides were placed in a petri dish containing medium for isolation and preservation of spermatozoa (1% M199 in HBSS with 20 mM HEPES buffer and 5% BSA) or Whitten’s Media (100 mM NaCl, 4.7 mM KCl, 1.2 mM KH_2_PO_4_, 1.2 mM MgSO_4_, 5.5 mM glucose, 1 mM pyruvic acid, and 4.8 mM lactic acid), depending on the subsequent analysis. Isolated epididymides were finely punctuated with a 25G needle to enable spermatozoa to be released into the medium, and incubated at 37°C for 10 min. Released spermatozoa were collected and centrifuged for 5 min at 700×*g* at room temperature. The supernatant was removed, and the pellet was resuspended in the appropriate medium depending on the subsequent analysis. Concentrations of isolated spermatozoa were calculated using a Makler counting chamber (Sefi-Medical Instruments, Ltd, Israel). Isolated spermatozoa were used for the capacitation and acrosome reaction procedure and the rest of the spermatozoa were stored at −70°C, before RNA isolation and the subsequent gene transcription analysis. To determine the spermatozoa functionality, approximately 1.5 × 10^5^ spermatozoa in 50 μl of Whitten’s Media were mixed with 350 μl of WH+ media [Whitten’s Media supplemented with the 10 mg/ml BSA (bovine serum albumin) and 20 mM of NaHCO_3_, to stimulate the capacitation] with a drop of mineral oil, at 37°C (5% CO_2_) for 1 h. Fifty microliters of capacitated spermatozoa was transferred into two new tubes, one without the progesterone, present as the control of the acrosome reaction, and one with 15 μM progesterone (PROG) to activate the acrosome reaction, with a drop of mineral oil, and incubated at 37°C (5% CO_2_) for 30 min. For the fixation of spermatozoa after the acrosome reaction, 20 μl of the spermatozoa suspension from each tube was mixed with 100 μl of the fixation solution (20 mM Na_2_HPO_4_, 150 mM NaCl, and 7.5% formaldehyde) and incubated for 20 min at room temperature. Subsequently, fixed spermatozoa were centrifuged for 1 min at 12,000×*g*, and washed with 100 mM ammonium acetate, pH 9. Smears of fixed spermatozoa were prepared on microscopic slides and air-dried. Dried spermatozoa smears were stained using staining solution (0.04% Coomassie Blue–G250, 50% methanol and 10% acetic acid) for 5 min at room temperature. Staining solution was rinsed with distilled water and spermatozoa smears were allowed to air-dry. Stained smears were analyzed using the Leica DMLB 100T microscope with 1,000× magnification, and up to 100 spermatozoa per slide were counted to determine the acrosomal status. Blue staining in the acrosomal region of the head indicated intact acrosome, while spermatozoa without blue staining in the acrosomal region were considered to be acrosome-reacted. Data are presented as the percentage of acrosome-reacted spermatozoa ± SEM.

### Isolation of RNA and cDNA Synthesis

Total RNA isolation was performed using the GenElute Mammalian Total RNA Miniprep Kit according to the protocol recommended by the manufacturer, followed by the DNase I (RNase-free) treatment. The first-strand cDNA was synthesized using the High-Capacity Kit for cDNA preparation.

### Relative Quantification of Gene Expression

Rat spermatozoa samples isolated from caudal epididymides were stored at −70°C until they were used for the isolation of total RNA. Total RNA isolation was performed using the GenElute Mammalian Total RNA Miniprep Kit according to the protocol recommended by the manufacturer (Sigma Aldrich, Germany, https://www.sigmaaldrich.com). To eliminate DNA from the samples, DNase I (RNase-free) treatment was carried out according to the manufacturer’s instructions (New England Biolabs, Massachusetts, United States, https://www.neb.com). The concentration and purity of isolated total RNA were measured using the BioSpec-nano spectrophotometer (Shimadzu, Japan, https://www.shimadzu.com). Furthermore, the first-strand cDNA was synthesized using the High Capacity Kit for cDNA preparation according to the manufacturer’s protocol (Thermo Fisher Scientific, Massachusetts, United States, https://www.thermofisher.com). In each set of reactions, negative controls consisting of non-reverse-transcribed samples were included. Quality of RNA and DNA integrity was checked using control primers for *Gapdh*, as described previously by our group [for references, please see (10, 11, 47)]. Relative expression of genes was quantified by real-time PCR (RQ-PCR) using SYBR Green-based chemistry from Applied Biosystems. Each reaction contained 10 ng of cDNA (calculated from starting RNA) in a volume of 2.5 μl and specific primers at a final concentration of 500 nM. Primer sequences used for RQ-PCR analysis, average Ct values, as well as GenBank accession codes for full gene sequences are given in **Supplementary Tables S5–S11**. Relative gene expression quantification of *Gapdh* was measured in each sample and used to correct variations in cDNA content between samples. Relative quantification of each gene was performed in duplicate, three times for each sample of two independent *in vivo* experiments. The real-time PCR reactions were carried out in the Eppendorf Mastercycler ep realplex 4 Real Time PCR and post-run analyses were performed using Mastercycler ep realplex Software. The heat map image was generated with relative fold change values, using the online tool CIMminer (http://discover.nci.nih.gov/cimminer/home.do as of December 13, 2021) to represent the gene expression profile of mitochondrial dynamic and functionality markers and signaling molecules regulating mitochondrial dynamics and functionality in different time points after the acute immobilization stress.

### Relative Quantification of Protein Expression and Immunoprecipitation Analysis

Rat spermatozoa samples isolated from caudal epididymides were frozen and stored at −70°C until protein extraction. Cells were lysed and Western blot analysis was performed as described previously (9). Immune-reactive bands were detected using MyECL Imager (Thermo Fisher Scientific Inc.; https://www.thermofisher.com) and analyzed as two-dimensional images using Image J version 1.48 (http://rsbweb.nih.gov/ij/download.html). The optical density of images is expressed as volume adjusted for the background, which gives arbitrary units of adjusted volume. Normalization of the data was done using GAPDH protein expression as the endogenous control. Immune detection was performed with different antibodies (all details are listed in **Supplementary Table S12**). Antibodies against PGC1, NRF1, NRF2, and GAPDH were purchased from Santa Cruz Biotechnology (https://www.scbt.com).

Spermatozoa samples for immunoprecipitation analysis were lysed in 1 ml of buffer containing 20 mM HEPES, 10 mM EDTA, 2.5 mM MgCl_2_, 40 mM β-glycerophosphate, 1 mM DTT, 1% NP-40, 0.5 mM 4-(aminoethyl)-benzenesulfonyl fluoride hydrochloride, 1 µM aprotinin, 2 µM leupeptin, and phosphatase inhibitor cocktail tablets [cont. (1R, 2S, 3R, 6S)-1.2-dimethyl-3.6-epoxycyclohexane-1.2-dicarboxylic anhydride]. The concentration of proteins in each sample was estimated by the Bradford method and set at a concentration of 300 µg/ml. An equal amount of protein in each sample (300 µg) was used for the immunoprecipitation. Pre-clearing of the lysate was done using 5 µl of normal goat serum [Santa Cruz Biotechnology, normal goat serum: sc-2043, (https://www.scbt.com)] mixed with 1 ml of lysate and incubated on ice for 1 h. After the incubation, 100 µl of bead slurry was added to each sample and incubated for 30 min at 4°C with gentle agitation. The supernatant for the immunoprecipitation was collected after 10 min and centrifuged at 14,000×*g* at 4°C. After the pre-clearing process, lysates were mixed with MFN2 antibody (Santa Cruz Biotechnology) and incubated at 4°C overnight with constant rotation. During additional overnight incubation at 4°C with constant rotation, immunoprecipitated complexes with MFN2 antibody were recovered by 80 µl of protein G agarose bead slurry. Precipitated proteins were washed two times with 1 ml of lysis buffer and the supernatant was used for further protein analysis (please see **Supplemental Material file**). Final pellets were mixed with protein loading dye, incubated at 100°C for 5 min, and resuspended in the SDS-PAGE 12% gels. Gels were analyzed by one-dimensional SDS-PAGE and proteins were transferred to a polyvinylidene difluoride membrane using a wet transfer. The immunodetection of the MFN2, PRKAc, and p38 MAPK was done with the use of MFN2 antibody (Santa Cruz Biotechnology), PRKAc antibody (BD Transductions Laboratories), and p38 MAPK antibody (Cell Signaling Technology) (all details are listed in **Supplementary Table S12**). Immune-reactive bands were detected using MyECL Imager (Thermo Fisher Scientific Inc.; https://www.thermofisher.com) and analyzed as two-dimensional images using Image J version 1.48 (http://rsbweb.nih.gov/ij/download.html). The optical density of images is expressed as volume adjusted for the background, which gives arbitrary units of adjusted volume. Normalization of the data was done using MFN2 protein expression.

### Statistical Analysis

Results of the experiments represent group means ± SEM values of the individual variation from two independent experiments. In each experiment, both control and stressed animals were randomly divided into four time point groups, with a total of 4 animals in the control group and 6 animals in the 1x3hIMO+R group per time point. In each of the two experiments, both control and stress animals were randomly divided into four time-points groups. In the first experiment, spermatozoa samples of each individual animal were used for the RNA extraction, individual cDNA, individual real-time PCR for the analysis of relative expression of transcripts, individual for protein extraction. While in the second experiment spermatozoa sample of each animal were pooled. Results from each experiment were analyzed by Mann–Whitney’s unpaired nonparametric two-tailed test (between the 1x3hIMO group and the control group within the same time point), or by one-way ANOVA followed by Dunnett’s test, for comparison with the ZT3-Control group. All statistical analyses were performed using GraphPad Prism 5 Software (GraphPad Software 287 Inc., La Jolla, CA, USA). In all cases, *p*-value <0.05 was considered to be statistically significant.

### Principal Component Analysis

Principal component analysis (PCA) was done with the dudi.PCA() function implemented in “ade4” package (49), on scaled and centered data matrix, within the R environment. We decided to retain the first two PCs based on eigenvalues and cumulative variation. In support of such a decision, we performed Horn’s parallel analysis for a PCA with the “paran” package, to adjust for finite sample bias in retaining components (50). Biplot visualization were performed with the “factoextra” package (51).

## Results

In order to properly understand the connection between acute stress, the most common stress in human society, and male (sub/in)fertility, the immobilization (IMO) stress of 3 h once (1x3hIMO) was applied to the adult male rats (11). The stress period was followed by recovery periods. Immediately after acute stress (ZT3) as well as 8 (ZT11), 14 (ZT17) and 20 (ZT23) h after acute stress, the number and functionality of spermatozoa, as well as the transcriptional profiles of 22 mitochondrial dynamics and function markers and 22 signaling molecules regulating both spermatozoa number/function and mitochondrial dynamics were tracked (**Figures 1**–**13**).

### Spermatozoa Number Is Lower 14 and 20 h After Acute Stress, While Functionality Declines Immediately After the Stress and 8 h Later, But Recovers 14 and 20 h After the Stress

The number of spermatozoa (**Figure 2A**) declined in rats having longer recovery periods, i.e., from the ZT17-1x3hIMO+R group (1.5-fold compared to ZT17-Control and 1.6-fold vs. ZT3-Control) and the ZT23-1x3hIMO+R group (2.9-fold compared to ZT23-Control and 2.6-fold vs. ZT3-Control). In contrast, the spermatozoa functionality (positive acrosome reaction) (**Figure 2B**) declined in groups of rats having shorter recovery periods, i.e., from the ZT3-1x3hIMO group (3.6-fold compared to ZT3-Control) and the ZT11-1x3hIMO+R group (1.4-fold compared to ZT11-Control and 1.5-fold vs. ZT3-Control).

In search for the possible mechanism(s) beyond these effects, the transcriptional profiles of mitochondrial dynamics markers and signaling molecules regulating both mitochondrial dynamics and spermatozoa number and functionality (important for fertilization) were tracked. Results showed that stress dramatically disturbed expression of transcripts for markers of mitochondrial dynamics and functionality as well as associated signaling pathways in spermatozoa. Expression levels of 40 out of 44 (91%) markers were changed either using ZT3-Control as a calibrator (**Figures 3**–**9**) or using the corresponding control at a particular ZT time point (**Supplementary Figures S1–S7**).

### Significant Changes in Transcriptional Profiles of Mitochondrial Dynamics and Functionality Markers in Spermatozoa From Acutely Stressed Rats Are Evident Up to 20 h After Stress

The transcriptional profiles of molecular markers of mitochondrial dynamics and functionality in spermatozoa are disturbed by acute stress since the transcriptional levels of 20 out of 22 (91%) markers were changed (**Figures 3**–**7** and **Supplementary Figures S1–S5**).

Mitochondrial biogenesis markers changed 8 out of 8 (100%). The level of transcripts for genes encoding PGC1 (*Ppargc1a* and *Ppargc1b*), very well known as the master regulator involved in the transcriptional control of all the processes related to mitochondrial homeostasis and integrator of environmental signals (32, 33), was disturbed (**Figure 3**). Interestingly, a circadian-like profile was observed in the expression of the *Ppargc1a* transcript since it was differently changed in spermatozoa taken from undisturbed rats at different time points: increased in the ZT11-Control (2.6-fold) and ZT17-Control (2.9-fold) groups compared to ZT3-Control, but decreased in ZT23-Control (2.1-fold). Changes were also detected in spermatozoa obtained from acutely stressed rats with different recovery periods: decrease in ZT11-1x3hIMO+R group (4.8-fold compared to ZT11-Control), but increase in ZT17-1x3hIMO+R (2.4-fold compared to ZT3-Control) and ZT23-1x3hIMO+R (3.6-fold compared to ZT23) groups. Less prominent effects were observed on the transcription of *Ppargc1b*: increased in spermatozoa from the ZT17-Control group (1.7-fold compared to ZT3-Control) and in the ZT23-1x3hIMO+R group (1.4-fold compared to ZT23-Control).

Transcription profiles of PGC1 downstream targets (*Nrf1*, *Nrf2a*, *Tfam*, *mtNd1*, and *Ppard*) that regulate genes for subunits of the oxidative phosphorylation (OXPHOS) also changed.

*Tfam* transcription was disturbed only at ZT23: decreased in the ZT23-Control group (2.3-fold compared to ZT3-Control), but increased in the ZT23-1x3hIMO+R group (3.7-fold compared to ZT23-Control and 1.6-fold vs. ZT3-Control).

*Nrf1* transcript decreased in spermatozoa from ZT23-Control (2.6-fold compared to ZT3-Control), but increased in all stressed groups with recovery period: ZT11-1x3hIMO+R (1.5-fold vs. ZT11-Control, 1.7-fold vs. ZT3-Control group), ZT17-1x3hIMO+R (1.5-fold vs. ZT17-Control), and ZT23-1x3hIMO+R (4.9-fold vs. ZT23-Control, 2.0-fold vs. ZT3-Control).

*Nrf2a* transcription was less disturbed than *Nrf1*: increased in ZT11-1x3hIMO+R (1.6-fold compared to ZT11-Control, 1.8-fold vs. the ZT3-Control group) and ZT23-1x3hIMO+R (2.7-fold vs. ZT23-Control, 1.9- fold vs. ZT3-Control) groups.

*Ppara* transcription profile increased in spermatozoa obtained from rats of all control groups compared to ZT3-Control (1.8-fold in ZT11-Control, 2.5-fold in ZT17-Control, 2.8-fold in ZT23-Control). In spermatozoa from stressed animals, an increased level of *Ppara* transcript was observed in ZT11-1x3hIMO+R (2.0-fold vs. ZT3-Control group) and ZT17-1x3hIMO+R (2.5-fold vs. ZT3-Control) groups, but *Ppara* transcript decreased in the ZT23-1x3hIMO+R group (5.5-fold vs. ZT23-Control, 1.95-fold vs. ZT3-Control).

*Ppard* transcription was less disturbed than *Ppara* since change/decrease was observed only in the spermatozoa from ZT23-Control (2.1-fold) compared to ZT3-Control. Increased *Ppard* levels were registered in spermatozoa of all stressed groups: ZT3-1x3hIMO+R (1.8-fold vs. ZT3-Control); ZT11-1x3hIMO+R (2.0-fold vs. ZT11, 1.6-fold vs. ZT3-Control), ZT17-1x3hIMO+R (2.1-fold vs. ZT-17, 2.1-fold vs. ZT3-Control), and ZT23-1x3hIMO+R (4.7-fold vs. ZT-23, 2.3-fold vs. ZT3-Control).

***mtNd1*** transcription profile was similar to *Ppargc1a*. Increased *mtNd1* level was detected in spermatozoa from ZT11-Control (1.6-fold) and ZT17-Control (1.9-fold) groups compared to ZT3-Control, but *mtNd1* decreased in ZT23-Control (4.5-fold). Changes were also detected in spermatozoa obtained from acutely stressed rats with different recovery periods: decreased in the ZT11-1x3hIMO+R group (1.5-fold compared to ZT11-Control), but increased in the ZT17-1x3hIMO+R (1.5-fold compared to ZT3-Control) and ZT23-1x3hIMO+R (5.0-fold compared to ZT23) groups.

Mitochondrial fusion markers changed 3 out of 3 (100%). Changes in transcriptional profiles of all spermatozoal mitofusion as well as mito-architecture markers (*Mfn1*, *Mfn2* and *Opa1*) were observed at the ZT23 time point (**Figure 4A**).

*Mfn1* transcription decreased in spermatozoa from the ZT23-Control group (3.9-fold vs. ZT3-Control), but increased in the ZT23-1x3hIMO+R group (8.1-fold compared to ZT23 control, 2.1-fold vs. ZT3-Control).

*Mfn2* transcription profile was similar to *Mfn1*. The level of *Mfn2* transcript decreased in spermatozoa from the ZT23-Control group (4.3-fold vs. ZT3-Control), but increased in the ZT23-1x3hIMO+R group (6.4-fold compared to ZT23 control, 1.5-fold vs. ZT3-Control).

*Opa1* transcript profile was similar to *Mfn1* and *Mfn2*. The level of *Opa1* transcript significantly decreased in spermatozoa obtained from the ZT23-Control group (2.7-fold vs. ZT3-Control), but increased in the ZT23-1x3hIMO+R group (5.3-fold compared to ZT23-Control, 1.9-fold vs. ZT3-Control).

Considering the importance of MFN2 expression for spermatozoa motility and viability as well as mitochondrial network homeostasis (31), interactions of MFN2 and proteins regulating both mitochondrial dynamic and spermatozoa number and functionality were followed. Results show that MFN2 protein interacts with the PRKA catalytic subunit in spermatozoa, but there is no significant difference in spermatozoa of the 1x3hIMO+R group compared to the ZT3-Control group. Immunoprecipitation analysis of MFN2 followed by Western blot analysis with p38 MAPK protein shows that the interaction between these proteins exists in spermatozoa, and that there is significant decrease in the control group of ZT17 and ZT23, as well as in the 1x3hIMO+R group of the ZT23 time point, compared to the ZT3-Control group (**Figure 4B**).

Mitochondrial fission markers changed 2 out of 2 (100%). Levels of transcripts for *Drp1* and *Fis1* differently changed at different the ZT time points (**Figure 5**).

*Fis1* transcription increased in spermatozoa from ZT17-Control (1.9-fold compared to ZT3-Control). In spermatozoa from stressed rats, a decrease was observed in the ZT17-1x3hIMO+R group (1.5-fold vs. ZT17-Control) and the opposite effect (increase) was detected in the ZT23-1x3hIMO+R group (1.8-fold vs. ZT23-Control, 2.4-fold vs. ZT3-Control).

*Drp1* transcript profile was different from *Fis1*, since changes/decreases were evident only in stressed groups: ZT11-1x3hIMO+R (2.8-fold vs. ZT11-Control) and ZT17-1x3hIMO+R groups (1.8-fold vs. ZT17-Control).

Mitochondrial autophagy markers changed 1 out of 3 (33%). Significant changes were evident only on the transcription profile of *Prkn*: decrease in ZT17-Control (3.8-fold compared to ZT3-Control) and ZT17-1x3hIMO+R (5.3-fold vs. ZT3-Control) groups. The transcriptional profile of *Pink1* and *Tfeb* remained unchanged (**Figure 6**).

Mitochondrial functionality markers changed 6 out of 6 (100%). Transcriptional profiles of NRF1/NRF2 downstream targets (CytC, COX4, and UCPs) serving as mitochondrial functional markers as well as the mediators of regulated proton leak and controllers of the production of superoxide and other downstream reactive oxygen species (41) were significantly changed at the ZT23 time point (**Figure 7**).

*Cox4i1* transcription significantly decreased in spermatozoa from the ZT23-Control group (3.4-fold vs. ZT3-Control) but increased in the ZT23-1x3hIMO+R group (6.1-fold compared to ZT23-Control, 1.8-fold vs. ZT3-Control).

*Cox4i2* transcript level significantly decreased in spermatozoa from the ZT23-Control (2.4-fold vs. ZT3-Control) as well as the stressed groups ZT17-1x3hIMO+R (1.6-fold compared to ZT17-Control, 1.9-fold vs. ZT3-Control) and ZT23-1x3hIMO+R (2.4-fold compared to ZT3-Control).

*Cytc* transcription significantly increased only in spermatozoa obtained from the ZT23-1x3hIMO+R group (1.8-fold compared to ZT23-Control).

*Ucp1* transcript level was significantly lower only in spermatozoa obtained from the ZT23-1x3hIMO+R group (8.9-fold compared to ZT23-Control, 4.3-fold vs. ZT3-Control).

*Ucp2* transcript in spermatozoa (the most abundantly expressed UCP gene in spermatozoa; *Ucp2*-Ct=22.07>*Ucp3*-Ct=29.81>*Ucp1*-Ct=29.96) was changed at ZT17 and ZT23 time points. *Ucp2* transcript level increased in spermatozoa isolated from ZT17-Control (1.8-fold compared to ZT3-Control), but decreased in spermatozoa from ZT23-Control (1.7-fold vs. ZT3-Control). In spermatozoa from stressed rats, increase was detected in ZT17-1x3hIMO+R (1.8-fold vs. ZT3-Control) and ZT23-1x3hIMO+R (3.4-fold compared to ZT23-Control, 2.0-fold compared to ZT3-Control).

*Ucp3* transcription increased in spermatozoa from ZT11-Control (1.6-fold compared to ZT3-Control) as well as ZT23-Control (2.7-fold vs. ZT3-Control), but decreased in ZT23-1x3hIMO+R (20.8-fold compared to ZT23-Control, 7.7-fold compared to ZT3-Control).

The results of the PCA confirmed that separation of the effects of acute stress recovery on mitochondrial dynamics marker elements depends on the day phase. It is clear that the transcriptional patterns were different during the active and inactive phases. Most of the transcripts were highly expressed during the active phase, which is expected given that stress occurred at the beginning of the inactive phase. Expression of the transcripts for proteins involved in mitochondrial dynamics tends to separate across the first two PCs for 74.2% of the total dataset of mitochondria-related gene variability. Also, the results offer different acute stress recovery effects on transcript expression: a pronounced cluster of genes encoding the elements essential for mitochondrial dynamics in the active phase opposes *Ucp3, Ppara*, and *Ucp1* in the inactive phase (**Figure 12A**, variable loadings are shown in **Supplementary Table S1**).

Since the cAMP and MAPK signaling are crucial not only for the regulation of spermatozoa number and functionality (43), but also for the regulation of mitochondrial dynamics and functionality (32, 33, 37), the transcriptional profiles of main signaling molecules were tracked.

### Significant Changes in Transcriptional Profiles of Signaling Molecules Regulating the Number and Functionality of Spermatozoa, as Well as the Mitochondrial Dynamics and Functionality in Spermatozoa From Stressed Rats Are Evident Up to 20 h After Stress

Markers of signaling pathways regulating the spermatozoa number/functionality as well as mitochondrial dynamics/functionality, both very important for male fertility, significantly changed during the recovery time course. Transcriptional levels of 20 out of 22 (91%) markers were changed, and most of the changes were increases of the expression (**Figures 8**, **9** and **Supplementary Figures S6** and **S7**).

cAMP signaling markers changed 11 out of 12 (92%). Most of the changes in transcriptional profile of cAMP signaling markers during stress recovery time periods were increased expression of most of the adenylyl cyclases (*Adcy3*, *Adcy5, Adcy6*, and *Adcy7*) except for *Adcy8* (decreased), *Adcy7* (remained unchanged), and *Adcy10* (decreased). In the same spermatozoa samples, the level of the transcripts for all genes encoding the catalytic and the regulatory protein kinase A subunits (*Prkaca, Prkacb, Prkar1a, Prkar2a*, and *Prkar2b*) increased (**Figure 8**).

*Adcy3* transcript levels increased in spermatozoa isolated from ZT11-Control (2.4-fold compared to ZT3-Control) and ZT17-Control (2.7-fold vs. ZT3-Control). In spermatozoa from stressed rats, increases were detected in ZT11-1x3hIMO+R (2.8-fold vs. ZT3-Control), ZT17-1x3hIMO+R (1.5-fold vs. ZT3-Control), and ZT23-1x3hIMO+R (4.5-fold compared to ZT23-Control, 4.5-fold compared to ZT3-Control).

*Adcy5* transcriptional profile was changed/increased only in spermatozoa from the stressed rats with recovery for 14 or 20 h: ZT17-1x3hIMO+R (1.7-fold vs. ZT17-Control) and ZT23-1x3hIMO+R (1.9-fold compared to ZT23-Control, 1.8-fold compared to ZT3-Control).

*Adcy6* transcription increased in spermatozoa isolated from ZT11-Control (1.7-fold compared to ZT3-Control), as well as from ZT23-1x3hIMO+R (2.5-fold compared to ZT23-Control, 1.6-fold compared to ZT3-Control). The opposite changes (decreased expression) were detected in ZT3-1x3hIMO+R (1.9-fold vs. ZT3-Control) and ZT11-1x3hIMO+R (1.4-fold vs. ZT11-Control).

*Adcy7* transcript levels decreased in spermatozoa from ZT23-Control (1.6-fold compared to ZT3-Control). In contrast, increased expressions were observed in the stressed group at all recovery time points: ZT11-1x3hIMO+R (2.0-fold vs. ZT11-Control), ZT17-1x3hIMO+R (2.1-fold vs. ZT17-Control), and ZT23-1x3hIMO+R (4.3-fold compared to ZT23-Control, 2.7-fold compared to ZT3-Control).

*Adcy8* transcription increased in spermatozoa isolated from ZT23-Control (1.7-fold compared to ZT3-Control). In spermatozoa from the stressed rats, decreases were detected in ZT3-1x3hIMO+R (2.3-fold vs. ZT3-Control), ZT17-1x3hIMO+R (2.4-fold vs. ZT17-Control, 1.7-fold vs. ZT3-Control), and ZT23-1x3hIMO+R (188.9-fold compared to ZT23-Control, 111.1-fold compared to ZT3-Control).

*Adcy10* transcriptional profile was changed/decreased only in spermatozoa from the stressed rats at all recovery time points: ZT11-1x3hIMO+R (1.5-fold vs. ZT11-Control), ZT17-1x3hIMO+R (1.4-fold vs. ZT17-Control), and ZT23-1x3hIMO+R (2.5-fold vs. ZT23-Control, 2.5-fold vs. ZT3-Control).

*Prkaca* transcription was changed only at the ZT23 time point: decreased in spermatozoa from the ZT23-Control group (2.8-fold compared to ZT3-Control), but increased in spermatozoa from the ZT23-1x3hIMO+R group (4.1 fold compared to ZT23-Control, 1.5 fold compared to ZT3-Control).

*Prkacb* transcription profile was similar to *Prkaca*. The level of *Prkacb* transcripts decreased in spermatozoa from the ZT23-Control group (2.6-fold compared to ZT3-Control), but increased in the ZT23-1x3hIMO+R group (5.8-fold compared to ZT23-Control, 2.2-fold vs. ZT3-Control).

*Prkar1a* transcription decreased in spermatozoa isolated from ZT23-Control (3.8-fold compared to ZT3-Control). In contrast, transcription increased in the ZT11-1x3hIMO+R (1.5-fold vs. ZT11-Control) and the ZT23-1x3hIMO+R (4.6-fold compared to ZT23-Control) groups.

*Prkar2a* transcriptional profile was similar to the profiles of transcripts for catalytic subunits of PRKA: decreased in spermatozoa from the ZT23-Control group (2.3-fold compared to ZT3-Control) but increased in the ZT23-1x3hIMO+R group (3.7-fold vs. ZT23-Control, 1.6-fold vs. ZT3-Control).

*Prkar2b* transcription profile was similar to *Prkar2a.* The level of *Prkar2b* transcripts decreased in spermatozoa from the ZT23-Control group (1.9-fold compared to ZT3-Control) but increased in the ZT23-1x3hIMO+R group (3.3-fold compared to ZT23-Control, 1.8-fold vs. ZT3-Control).

The results of the PCA confirmed the separation of the effects of acute stress recovery on cAMP signaling pathway elements. It is clear that the transcriptional patterns were different during the active and inactive phases and that the transcripts were highly expressed during the active phase. Most of the transcripts were highly expressed during the active phase, which is expected given that stress occurred at the beginning of the inactive phase. Expression of the transcripts for proteins involved in cAMP signaling accounts for 78.2% variability. Also, the results offer different acute stress recovery effects on the transcripts’ expression: a pronounced cluster of *Adcy8* and *Adcy10* in the inactive phase opposes gene clusters encoding the other elements of cAMP signaling in the active phase (**Figures 8**, **12B**, variable loadings are shown in **Supplementary Table S2**).

MAPK signaling markers changed 9 out of 10 (90%). The markers of MAPK signaling (*Mapk1, Mapk3, Mapk6, Mapk7, Mapk8, Mapk9, Mapk11, Mapk12, Mapk13*, and *Mapk14*) were affected at all time points and more than the above-mentioned markers in spermatozoa. Transcripts of all markers significantly increase, except for the decreased transcription of *Mapk11*, in spermatozoa isolated from the groups of rats exposed to acute stress for 3 h and recovered for 20 h, while most of the markers increased in spermatozoa from the stressed rats at all recovery time points (**Figure 9**).

*Mapk1* transcription decreased in spermatozoa isolated from ZT23-Control (2.7-fold compared to ZT3-Control). Increases were observed in spermatozoa from the stressed rats at all recovery time points: ZT3-1x3hIMO+R (1.9-fold vs. ZT3-Control), ZT11-1x3hIMO+R (2.0-fold vs. ZT11-Control, 2.0-fold vs. ZT3-Control), ZT17-1x3hIMO+R (1.4-fold vs. ZT17-Control, 1.8-fold vs. ZT3-Control), and ZT23-1x3hIMO+R (7.2-fold compared to ZT23-Control, 2.6-fold compared to ZT3-Control).

*Mapk3* transcript levels decreased in spermatozoa from the ZT23-Control group (2.3-fold compared to ZT3-Control) but increased in the ZT23-1x3hIMO+R group (4.1-fold compared to ZT23-Control, 1.8-fold vs. ZT3-Control).

*Mapk6* transcriptional profile was similar to *Mapk1*. The decline in the *Mapk6* transcription was observed in spermatozoa from ZT23-Control (2.4-fold compared to ZT3-Control). The significant increase was evident in spermatozoa from the stressed rats at all recovery time points: ZT3-1x3hIMO+R (1.6-fold vs. ZT3-Control), ZT11-1x3hIMO+R (2.8-fold compared to ZT11-Control, 1.8-fold vs. ZT3-Control), ZT17-1x3hIMO+R (2.1-fold vs. ZT17-Control; 1.7-fold vs. ZT3-Control), and ZT23-1x3hIMO+R (5.6-fold compared to ZT23-Control, 2.3-fold compared to ZT3-Control).

*Mapk8* transcriptional profile was similar to *Mapk1* and *Mapk6*, but the effect was absent at the ZT17 time point. The decreased *Mapk8* transcript level was evident in spermatozoa from ZT23-Control (2.5-fold compared to ZT3-Control). A significant increase was evident in spermatozoa from ZT3-1x3hIMO+R (1.4-fold compared to ZT3-Control), ZT11-1x3hIMO+R (1.5-fold compared to ZT11-Control), and ZT23-1x3hIMO+R (4.9-fold compared to ZT23-Control, 1.9-fold vs. ZT3-Control).

*Mapk9* transcription increased only in spermatozoa from the stressed rats recovered for 20 h, i.e., ZT23-1x3hIMO+R (3.0-fold vs. ZT23-Control, 1.9-fold compared to ZT3-Control).

*Mapk11* transcriptional profile was changed/increased only in spermatozoa from the stressed rats recovered for 14 and 20 h: ZT17-1x3hIMO+R (1.5-fold compared to ZT3-Control) and ZT23-1x3hIMO+R (2.1-fold compared to ZT23-Control).

*Mapk12* transcription significantly decreased in spermatozoa from ZT23-Control (3.3-fold compared to ZT3-Control). In spermatozoa from stressed rats, increases were detected in ZT11-1x3hIMO+R (2.0-fold compared to ZT11-Control, 1.8-fold compared to ZT3-Control) and ZT23-1x3hIMO+R (7.7-fold compared to ZT23-Control, 2.5-fold compared to ZT3-Control).

*Mapk13* transcript level significantly decreased in spermatozoa from ZT23-Control (2.7-fold compared to ZT3-Control). Significant increases were evident in spermatozoa from the rats recovered at different time points: ZT11-1x3hIMO+R (1.7-fold vs. ZT11-Control, 1.6-fold vs. ZT3-Control), ZT17-1x3hIMO+R (1.7-fold vs. ZT17-Control), and ZT23-1x3hIMO+R (3.4-fold vs. to ZT23-Control).

*Mapk14* transcription was changed only at the ZT23 time point: decreased in spermatozoa from ZT23-Control (2.1-fold compared to ZT3-Control), but increased in spermatozoa from ZT23-1x3hIMO+R (4.3-fold compared to ZT23-Control, 2.1-fold compared to ZT3-Control).

The results of the PCA show significant separation of the effects of acute stress recovery on MAPK signaling pathway elements depending on the day phase. It is clear that the transcriptional patterns were different during the active and inactive phases and that the transcripts were highly expressed during the active phase (**Figures 9**, **12C** as well as **Supplementary Table S3**).

The results of the PCA confirmed the separation of the effects of acute stress recovery on MAPK signaling. The transcriptional patterns were different during the active and inactive phases. Most of the transcripts were highly expressed during the active phase, which is expected given that stress occurred at the beginning of the inactive phase. Expression of the transcript of the proteins involved in MAPK signaling accounts for 82.1% of data variability. The results offered different acute stress recovery effects on the transcript expression: a pronounced *Mapk11* in the inactive phase opposes clusters of other transcripts for elements of MAPK signaling (**Figures 9**, **12C**, variable loadings are shown in **Supplementary Table S3**).

For a better understanding, results showing the transcriptional profiles of mitochondrial dynamics and functionality markers and signaling molecules regulating mitochondrial dynamics and functionality as well as spermatozoa number and functionality in spermatozoa of stressed adult rats with different periods for recovery are summarized in **Table 1**.

**Table 1:** The transcriptional profiles of mitochondrial dynamics and functionality markers and signaling molecules regulating mitochondrial dynamics and functionality as well as spermatozoa number and functionality in spermatozoa of stressed adult rats.

Group Transcript	Time points							
	ZT3		ZT11		ZT17		ZT23	
	Control	1x3hIMO	Control	1x3hIMO	Control	1x3hIMO	Control	1x3hIMO
***Ppargc1a***	**1.0** ± 0.11	**1.0** ± 0.20	**2.6^#^** ± 0.54 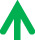	0.5* ± 0.14 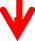	**2.9^#^** ± 0.82 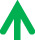	**2.4^#^** ± 0.59 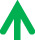	**0.5^#^** ± 0.18 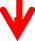	1.7* ± 0.65 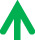
***Ppargc1b***	**1.0** ± 0.11	**0.6** ± 0.11	**0.8** ± 0.01	**0.8** ± 0.05	**1.7^#^** ± 0.32 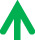	**1.2** ± 0.07	**0.8** ± 0.13	1.4* ± 0.03 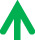
***Tfam***	**1.0** ± 0.10	**1.2** ± 0.13	**1.0** ± 0.05	**1.3** ± 0.02	**1.0** ± 0.04	**1.3** ± 0.01	**0.4^#^** ± 0.05 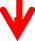	1.6*^#^ ± 0.01 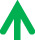
***Nrf1***	**1.0** ± 0.11	**1.1** ± 0.02	**1.1** ± 0.04	1.7*^#^ ± 0.18 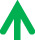	**0.8** ± 0.07	1.3* ± 0.03 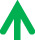	**0.4^#^** ± 0.01 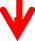	2.0*^#^ ± 0.12 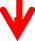
***Nrf2a***	**1.0** ± 0.11	**1.2** ± 0.07	**1.1** ± 0.05	1.8*^#^ ± 0.10	**1.2** ± 0.09	**1.4** ± 0.06	**0.7** ± 0.03	1.9*^#^ ± 0.12 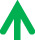
***Ppara***	**1.0** ± 0.11	**1.4** ± 0.29	**1.8^#^** ± 0.32 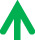	**2.0^#^** ± 0.16	**2.5^#^** ± 0.17 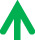	**2.5^#^** ± 0.10 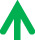	**2.8^#^** ± 0.36 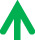	0.5*^#^ ± 0.08 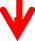
***Ppard***	**1.0** ± 0.12	1.8*^#^ ± 0.14 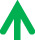	**0.8** ± 0.04	1.6*^#^ ± 0.03 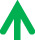	**1.0** ± 0.02	2.1*^#^ ± 0.00 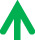	**0.5^#^** ± 0.01 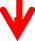	2.3*^#^ ± 0.04 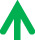
***mtNd1***	**1.0** ± 0.12	**1.1** ± 0.06	**1.6^#^** ± 0.01	1.1* ± 0.02 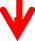	**1.9^#^** ± 0.04 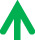	**1.5^#^** ± 0.04 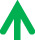	**0.2^#^** ± 0.01 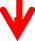	1.1* ± 0.03 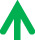
***Mfn1***	**1.0** ± 0.11	**1.0** ± 0.11	**0.9** ± 0.06	**1.2** ± 0.15	**1.0** ± 0.15	**1.3** ± 0.16	**0.3^#^** ± 0.02 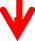	2.1*^#^ ± 0.12 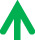
***Mfn2***	**1.0** ± 0.11	**0.8** ± 0.11	**0.7** ± 0.02	**0.9** ± 0.09	**0.7** ± 0.11	**0.9** ± 0.03	**0.2^#^** ± 0.03 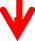	1.5*^#^ ± 0.10 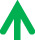
***Opa1***	**1.0** ± 0.11	**1.3** ± 0.01	**0.8** ± 0.05	**1.2** ± 0.05	**1.1** ± 0.01	**1.1** ± 0.06	**0.4^#^** ± 0.02 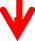	1.9*^#^ ± 0.01 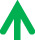
***Fis1***	**1.0** ± 0.11	**1.2** ± 0.12	**0.8** ± 0.10	**0.7** ± 0.06	**1.9^#^** ± 0.06 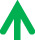	1.3* ± 0.06 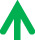	**1.3** ± 0.10	2.4*^#^ ± 0.39 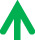
***Drp1***	**1.0** ± 0.11	**1.0** ± 0.13	**1.4** ± 0.04	0.5* ± 0.32 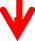	**1.1** ± 0.01	0.6* ± 0.02 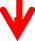	**1.3** ± 0.03	**1.5** ± 0.07
***Prkn***	**1.0** ± 0.05	**0.5** ± 0.22	**0.65** ± 0.15	**0.6** ± 0.10	**0.3^#^** ± 0.09 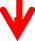	**0.2^#^** ± 0.05 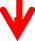	**0.5** ± 0.12	**0.7** ± 0.16
***Cox4i1***	**1.0** ± 0.12	**1.3** ± 0.10	**1.4** ± 0.07	**1.4** ± 0.07	**1.2** ± 0.10	**1.0** ± 0.12	**0.3^#^** ± 0.01 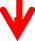	1.8*^#^ ± 0.04 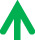
***Cox4i2***	**1.0** ± 0.12	**0.6** ± 0.27	**1.8** ± 0.45	**1.2** ± 0.31	**0.8** ± 0.01	0.5*^#^ ± 0.05 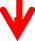	**0.4^#^** ± 0.03 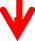	**0.4^#^** ± 0.02 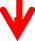
***Cytc***	**1.0** ± 0.12	**1.3** ± 0.05	**1.2** ± 0.04	**1.4** ± 0.05	**1.2** ± 0.02	**1.3** ± 0.01	**0.7** ± 0.01	1.4* ± 0.01 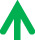
***Ucp1***	**1.0** ± 0.12	**1.4** ± 0.47	**2.1** ± 0.59	**1.2** ± 0.42	**2.5^#^** ± 0.48 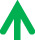	**2.3^#^** ± 0.88 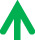	**2.1^#^** ± 0.71 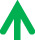	0.2*^#^ ± 0.04 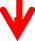
***Ucp2***	**1.0** ± 0.11	**1.2** ± 0.03	**1.1** ± 0.04	**1.3** ± 0.09	**1.8^#^** ± 0.02 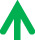	**1.8^#^** ± 0.04 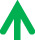	**0.6^#^** ± 0.01 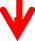	2.1*^#^ ± 0.07 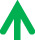
***Ucp3***	**1.0** ± 0.11	**0.8** ± 0.09	**1.6^#^** ± 0.21 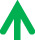	**1.2** ± 0.31	**1.0** ± 0.31	**1.0** ± 0.15	**2.7^#^** ± 0.19 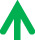	0.1*^#^ ± 0.02 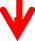
***Adcy3***	**1.0** ± 0.11	**1.1** ± 0.12	**2.4^#^** ± 0.13 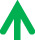	**2.8^#^** ± 0.27 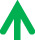	**0.9** ± 0.20	1.5* ± 0.43 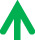	**1.1** ± 0.18	4.5*^#^ ± 0.77 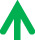
***Adcy5***	**1.0** ± 0.11	**1.3** ± 0.07	**1.0** ± 0.09	**1.3** ± 0.01	0.8 ± 0.09	**1.3** ± 0.07	**1.0** ± 0.22	**1.8** ± 0.18
***Adcy6***	**1.0** ± 0.11	**0.5** ± 0.01	**1.7^#^** ± 0.16 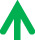	**1.2** ± 0.01	**1.4** ± 0.07	**1.5** ± 0.05	**0.6** ± 0.01	1.6*^#^ ± 0.04 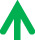
***Adcy7***	**1.0** ± 0.10	**1.2** ± 0.01	**0.7** ± 0.07 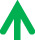	1.3* ± 0.14 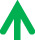	**0.8** ± 0.18	1.5* ± 0.05 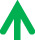	**0.6^#^** ± 0.03 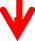	2.7*^#^ ± 0.13 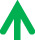
***Adcy8***	**1.0** ± 0.11	0.4* ± 0.13 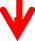	**1.1** ± 0.14	**1.2** ± 0.16	**1.4** ± 0.10	0.6*^#^ ± 0.03 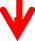	**1.7^#^** ± 0.14 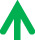	0.01*^#^ ± 0.0 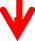
***Adcy10***	**1.0** ± 0.12	**0.9** ± 0.01	**1.4** ± 0.13	0.9* ± 0.08 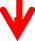	**1.3** ± 0.15	0.9* ± 0.08 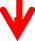	**1.0** ± 0.22	0.4*^#^ ± 0.14 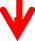
***Prkaca***	**1.0** ± 0.11	**0.7** ± 0.10	**1.3** ± 0.08	**1.0** ± 0.01	**1.2** ± 0.09	**1.1** ± 0.03	**0.4^#^** ± 0.03 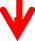	1.5*^#^ ± 0.09 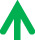
***Prkacb***	**1.0** ± 0.18	**0.9** ± 0.07	**1.3** ± 0.06	**1.5** ± 0.07	**1.0** ± 0.03	**1.0** ± 0.12	**0.4^#^** ± 0.01 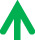	2.2*^#^ ± 0.2 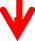
***Prkar1a***	**1.0** ± 0.10	**1.0** ± 0.08	**0.9** ± 0.04	1.4* ± 0.16 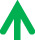	**1.0** ± 0.06	**1.2** ± 0.09	**0.3^#^** ± 0.01 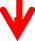	1.2* ± 0.02 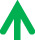
***Prkar2a***	**1.0** ± 0.13	**0.7** ± 0.09	**1.1** ± 0.09	**1.0** ± 0.06	**0.9** ± 0.07	**0.9** ± 0.07	**0.4^#^** ± 0.05 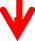	1.6*^#^ ± 0.14 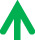
***Prkar2b***	**1.0** ± 0.09	**0.7** ± 0.07	**1.2** ± 0.10	**1.0** ± 0.09	**0.9** ± 0.01	**1.1** ± 0.09	**0.5^#^** ± 0.06 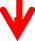	1.8*^#^ ± 0.05 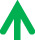
***Mapk1***	**1.0** ± 0.07	1.9*^#^ ± 0.11 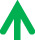	**1.0** ± 0.06	2.0*^#^ ± 0.15 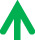	**1.2** ± 0.11	1.8*^#^ ± 0.04 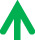	**0.4^#^** ± 0.01 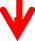	2.6*^#^ ± 0.16 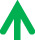
***Mapk3***	**1.0** ± 0.10	**0.7** ± 0.07	**1.1** ± 0.03	**1.2** ± 0.04	**1.1** ± 0.04	**1.0** ± 0.04	**0.4^#^** ± 0.06 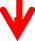	1.8*^#^ ± 0.04 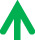
***Mapk6***	**1.0** ± 0.11	1.6*^#^ ± 0.13 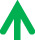	**0.6** ± 0.02	1.8*^#^ ± 0.04 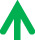	**0.8** ± 0.05	1.7*^#^ ± 0.03 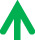	**0.4^#^** ± 0.04 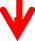	2.3*^#^ ± 0.04 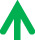
***Mapk8***	**1.0** ± 0.11	1.4*^#^ ± 0.11 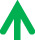	**1.2** ± 0.07	**1.5^#^** ± 0.07	**1.1** ± 0.04 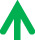	**1.3** ± 0.05	**0.4^#^** ± 0.05 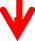	1.9*^#^ ± 0.29 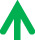
***Mapk9***	**1.0** ± 0.17	**1.0** ± 0.13	**1.1** ± 0.05	**1.2** ± 0.13	**1.0** ± 0.05	**1.2** ± 0.08	**0.6** ± 0.05	1.9*^#^ ± 0.14 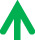
***Mapk11***	**1.0** ± 0.10	**0.8** ± 0.09	**1.3** ± 0.03	**1.2** ± 0.09	**1.2** ± 0.14	**1.5^#^** ± 0.07 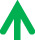	**1.4** ± 0.12	0.7* ± 0.10 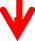
***Mapk12***	**1.0** ± 0.11	**1.0** ± 0.12	**0.9** ± 0.07	1.8*^#^ ± 0.09 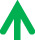	**1.2** ± 0.03	**1.4** ± 0.09	**0.3^#^** ± 0.04 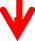	2.5*^#^ ± 0.15 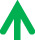
***Mapk13***	**1.0** ± 0.11	**0.6** ± 0.07	**1.6^#^** ± 0.16 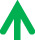	0.9* ± 0.06 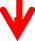	**1.3** ± 0.12	0.6* ± 0.29 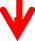	**0.4^#^** ± 0.01 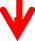	1.3* ± 0.05 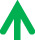
***Mapk14***	**1.0** ± 0.12	**0.8** ± 0.01	**1.0** ± 0.02	**1.3** ± 0.05	**1.1** ± 0.01	**1.1** ± 0.01	**0.5^#^** ± 0.01 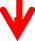	2.1*^#^ ± 0.01 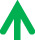

Data are presented as means ± SEM values of two independent experiments. Statistical significance at *p* < 0.05: * vs. the control group of each time point; ^#^ vs. the control group of ZT3 time point.

Green arrow indicates the increased level of the transcript, while red arrow indicates decreased level of the transcript.

## Discussion

It is very well known that life starts with fertilization. This process requires highly energizing and perfectly functioning spermatozoa. Unfortunately, many recent publications pointed to increased incidence of unexplained cases of (sub/in)fertility in men as well as a decrease in the fertility rate in men younger than age 30 (8, 12, 13). The semen quality and fertility are important not only as fundamental markers of reproductive health, but also as fundamental biomarkers of overall health (13, 52). Also, the World Health Organization (WHO) stated that the overall burden of infertility in men is high, unknown, and underestimated, and has not shown any decrease over the last 20 years. The WHO called for urgent investigations of the mechanisms of (sub/in)fertility (https://www.who.int/reproductivehealth/topics/infertility/perspective/en/).

In search for possible mechanisms of (sub/in)fertility as well as the connection between stress and male (sub/in)fertility, an *in vivo* model of acute psychological stress, the most common stress in human society, was applied on adult male rats and stress period was tracked with different recovery periods. Four time points were chosen (2 points during 12-h light/inactive phase and 2 points during 12-h dark/active phase): immediately after the 3-h acute stress (ZT3) as well as 8 (ZT11), 14 (ZT17), and 20 (ZT23) h later. The number/functionality (positive acrosome reaction) of spermatozoa and the transcriptional profiles of 22 mitochondrial dynamics/function markers and 22 related signaling molecules were tracked.

Results showed for the first time, to the best of our knowledge, that the acute stress-provoked effects appeared 20 h after the end of the stress, and this is very clearly shown on heat maps (**Figure 10** and **Supplementary Figure S8**). Lower number of spermatozoa was observed at ZT17 and ZT23, while decreased spermatozoa functionality (positive acrosome reaction) was evident at ZT3 and ZT11, but recovered at ZT17 and ZT23. Transcriptional profiles of 91% (20/22) of mitochondrial dynamics and functionality markers and 91% (20/22) of signaling molecules regulating both mitochondrial dynamics and spermatozoa number and functionality were disturbed after acute stress and during the recovery period (**Figures 10**, **11** and **Supplementary Figure S8**). The results of the PCA show the significant separation of effects of acute stress recovery during the active and inactive phase of the day. It is clear that the transcriptional patterns were different during the active and inactive phases and that most of the transcripts were highly expressed during the active/dark phase of the day (**Figure 12**). The physiological relevance is the recovered functionality (positive acrosome reaction), suggesting that molecular events are adaptive mechanism regulated by physiological stress response signaling. With this molecular scenario, the spermatozoa may try to preserve the basic mitochondrial network homeostasis and self-activity.

It is well known that stress signaling is involved in the regulation of spermatogenesis and fertility in a very complex and intriguing manner. Chronic intermittent stress irreversibly decreases sperm number (53–55) as well as sperm motility (56) and spermatozoa quality (57) in male rats. Our recently published articles showed that repeated psychophysical stress also lowered the number of spermatozoa (10, 11). The decline in progressively motile sperm in humans is associated with stress (58) and secondary infertility is significantly higher in patients with post-traumatic stress disorder (59). However, there are no published pieces of evidence related to the effects of stress recovery on spermatozoa number and functionality as well as signaling pathways associated with these processes. Here, we show that the number of epididymal spermatozoa declines 14 and 20 h after stress. It is difficult to give a precise explanation, but one of the reasons could be that mechanisms causing reduction in the number of spermatozoa started at earlier points, maybe as a consequence of stress hormone signaling activation, but they are visible at ZT17 and ZT20. Also, the reason could be, although not significantly, persistently higher levels of cortisol (48).

Since mitochondria are very important for many highly energy-driven processes including spermatozoa functionality and fertilization as well as stress response, it was of interest to follow the transcriptional profile of mitochondrial dynamics/functionality markers as well as signaling molecules regulating mitochondrial homeostasis and spermatozoa functionality. Results of transcriptional analyses clearly showed that effects of acute stress were visible up to 20 h later and most of the effects and prominent effects were observed at ZT23 (**Figures 10**, **11**). All those molecules are very important for spermatozoa functionality. Our results showed a circadian-like type of transcriptional profile of *Ppargc1a/*PGC1 in spermatozoa from both unstressed and stressed rats. It was published that PPARGC1A is changed in spermatozoa from patients suffering from type 2 diabetes mellitus (60) and that increased expression of *Nrf2* diminished testicular inflammation (61). Also, our preliminary results show protein interaction of PGC1 and NRF1 proteins in spermatozoa (data not shown). Moreover, expression of the TFAM gene correlates with sperm DNA fragmentation and mtDNA copy number (27, 28). Heat map analysis of the transcriptional profile of mitochondrial dynamics and functionality markers (**Figure 10A** and **Supplementary Figure 8A**) clearly showed that during recovery from acute stress, spermatozoa most abundantly express the main markers of mitochondrial fusion (*Mfn1, Mfn2*, and *Opa1*). This is very important for keeping the integrity of the mitochondrial network and energetic balance. These results may explain the findings of others showing the relation of the expression level of MFN2 to motility and cryoprotective potentials of human sperm (31, 62). Also, our results show for the first time, to the best of our knowledge, the interaction of MFN2 and the catalytic subunit of protein kinase A (PRKAc) in spermatozoa. This interaction was already confirmed in other cell types, with PRKA phosphorylation site at Serine 442 (63, 64). Presented results of immunoprecipitation analysis (MFN2/PRKAc) show no difference between the 1x3hIMO+R and the control group of all time points. On the other hand, results of immunoprecipitation analysis show an interaction between MFN2 and p38 MAPK proteins, with a decrease in ZT17-Control, ZT23-Control, and ZT23-1x3hIMO+R groups compared to the ZT3-Control group, suggesting that prolonged acute stress recovery influences the interaction between these two proteins in spermatozoa. Our results show the increased expression of transcript for *Cox4i1* in spermatozoa from stressed rats, the gene that encodes the terminal enzyme in the mitochondrial respiratory chain. It has been shown that this gene is also significantly increased in spermatozoa from obese males (65), and it is important for infertility treatment in men (66). Our results clearly show increased expression of transcript for *Ucp2* (most abundantly expressed UCP protein in rat spermatozoa) probably as a consequence of the stress hormone adrenaline (10). Our supplementary results show the trend of the increased mitochondrial membrane potential of spermatozoa treated with adrenaline (**Supplementary Figure S10**). These molecular events can increase spermatozoa motility since it was shown that UCP2 mitigates the loss of human spermatozoa motility (30). The results of the PCA show that most of the transcripts for the main markers of mitochondrial dynamics were highly expressed during the active/dark phase of the day (**Figure 12A**), suggesting the importance of molecular timing in regulation of the above-mentioned markers.

New insights into the understanding of molecular events related to the effects of acute stress on spermatozoa include our finding that shows that 91% of markers of signaling pathways regulating both mitochondrial dynamics and spermatozoa functionality are changed during the recovery from acute stress. Again, heat map analysis (**Figure 10B**, **Supplementary Figure S8**) clearly showed that changes are most abundant at the ZT23, and they are mostly increased expression. All increased transcripts are, for the signaling molecules, very well known as the essential regulators of spermatozoa number/functionality (43), as well as regulators of PGC1, the biogenesis of OXPHOS, mitofusion, mitofission, and mitophagy (32, 33, 37). Furthermore, all affected molecules are part of the complex signaling network in spermatozoa precisely regulated to provide fertility homeostasis in health and diseases (67). The consequences of the increased expression of transcripts are restored spermatozoa functionality at the ZT17 and ZT23 since it was shown that cAMP signaling improves sperm motility (68, 69) and it is important for the activation of CatSper channels (70). Increased expressions of transcripts for all subunits of PRKA are also a great adaptive and ameliorative mechanism since it was reported that the PRKAR2A reduction in asthenozoospermic patients decreases sperm quality (71), while Prkar2b is sensitive to heat (72). The results of the PCA clearly showed that the transcriptional patterns were different during the active and inactive phases and that most of the transcripts were highly expressed during the active/dark phase of the day. Interestingly, the transcript for the most important spermatozoal ADCY, ADCY10, was highly expressed during the inactive/light phase of the day (**Figure 12B**). Last, but not least, increased transcripts for *Mapk1*, *Mapk3*, and *Mapk14* in spermatozoa from rats recovered for 20 h could be compared with findings that testicular hyperthermia induces both MAPK1/3 and MAPK14 (73) and that MEK1/2 and ERK2 regulate the spermatozoa capacitation (74). The results of the PCA clearly showed that the transcriptional patterns of all analyzed MAPKs, except *Mapk11*, were highly expressed during the active/dark phase of the day (**Figure 12C**). Moreover, *Mapk8* significantly increased at ZT3 and ZT23, and it was shown that phosphorylation of MAPK8 is associated with germ cell apoptosis and redistribution of the Bcl2-modifying factor (75). All molecular events could be possible adaptive responses, and this was proven by recovery of functionality at ZT23.

We believe that results presented here have a significant translational aspect related to the effect of acute stress on male fertility. To prove that our results have translational significance, we started our analysis using spermatozoa obtained from human subjects, and preliminary results showed the correlation of different transcriptional profiles of the mitochondrial dynamics markers and different types of spermiograms. Moreover, according to the questionnaire completed by 115 patients from a governmental ART clinic providing the IVF service for free, 105/115 (91%) reported some degree of stress: 51/115 (44%) reported a low degree of stress, 41/115 (36%) reported frequent stressful situations, and 13/115 (11%) reported a high degree of stress (Tomanic et al., unpublished results).

It is important to point out that our investigation did not consider a possible contribution of epididymal cells to the RNA isolated from spermatozoa. This is important since spermatozoa RNA is subjected to epididymal RNA contamination that is transferred to spermatozoa *via* extracellular vesicles such as epididymosomes. Also, the aim of our study was not to assess motility, but certainly it is well known that mitochondria produce energy for sperm movement. Last, but not least, it is shown that structural abnormalities and decreased spermatozoa motility are associated with decrease in mitochondrial activity and decrease in basal oxygen consumption (76). Since stress stimulates reactive oxygen species and higher concentrations of reactive oxygen species can have detrimental effects on quality of spermatozoa (77), our results showing the increase in the expression of NRF1 and NRF2 transcripts and proteins could be the possible mechanism of adaptation for the restored spermatozoa functionality 20 h after acute stress.

Maybe it is noteworthy that all molecules involved in the regulation of spermatozoa homeostasis and functionality could be possible candidates and eventually responsible for male (in/sub)fertility. Recently, it was described that the sperm-specific form of lactate dehydrogenase is required for fertility and is an attractive target for male contraception (78). Since the existing literature suggests the importance of semen quality and male fertility not only as the fundamental marker of reproductive health but also as fundamental biomarkers of overall health and harbingers for the development of comorbidity and mortality, we anticipate our results to be a starting point for more investigations considering the mitochondrial dynamics markers, or their transcriptional profiles as possible predictors of (in/sub)fertility.

## Conclusions

Acute stress, the most common stress in human society, significantly changes 91% of followed mitochondrial dynamics and functionality markers as well as 91% of signaling molecules regulating spermatozoa homeostasis and mitochondrial dynamics/functionality. This leads to the recovery of spermatozoa number/functionality (positive acrosome reaction), which is important for male (in/sub)fertility. Stress-triggered changes represent adaptive mechanisms to keep spermatozoa functionality, and they are essential for fertility. Besides the effects of stress recovery, our results show the circadian-like nature in the expression of some important regulators of spermatozoa function.

## Data Availability Statement

The original contributions presented in the study are included in the article/**Supplementary Material**. Further inquiries can be directed to the corresponding author.

## Ethics Statement

All experimental protocols were approved (statement no. 01-201/3) by the local Ethical Committee on Animal Care and Use of the University of Novi Sad.

## Author Contributions

IS—acquisition of the data; analysis and interpretation of the data; drafting of the manuscript; revising manuscript critically for important intellectual content; final approval of the version to be submitted. SR.—acquisition of the data; analysis and interpretation of the data; revising manuscript critically for important intellectual content; final approval of the version to be submitted. TT—acquisition of the data; revising manuscript critically for important intellectual content; final approval of the version to be submitted. MM—acquisition of the data; revising manuscript critically for important intellectual content; final approval of the version to be submitted. TK—acquisition of the data; analysis and interpretation of the data; revising manuscript critically for important intellectual content; final approval of the version to be submitted. SA—the conception and design of the research; acquisition of the data; analysis and interpretation of the data; drafting the manuscript; revising manuscript critically for important intellectual content; final approval of the version to be submitted. All authors—approved the final version of the manuscript; agree to be accountable for all aspects of the work in ensuring that questions related to the accuracy or integrity of any part of the work are appropriately investigated and resolved; qualify for authorship, and all those who qualify for authorship are listed.

## Funding

This research was funded by the Serbian Ministry of Education, Science and Technological Development (CIV-CeRES-2021, 451-03-9/2021-14/200125) and the Autonomous Province of Vojvodina (2708).

## Conflict of Interest

The authors declare that the research was conducted in the absence of any commercial or financial relationships that could be construed as a potential conflict of interest.

## Publisher’s Note

All claims expressed in this article are solely those of the authors and do not necessarily represent those of their affiliated organizations, or those of the publisher, the editors and the reviewers. Any product that may be evaluated in this article, or claim that may be made by its manufacturer, is not guaranteed or endorsed by the publisher.

## Glossary

**Table:**

1x3hIMO	one time immobilization stress with a duration of 3 h
Adcy3	gene encoding adenylate cyclase 3
Adcy5	gene encoding adenylate cyclase5
Adcy6	gene encoding adenylate cyclase 6
Adcy7	gene encoding adenylatecyclase 7
Adcy8	gene encoding adenylate cyclase 8
Adcy10	gene encodingadenylate cyclase 10
COX4	cytochrome c oxidase subunit 4
Cox4i1	geneencoding cytochrome c oxidase subunit 4i1
Cox4i2	gene encoding cytochromec oxidase subunit 4i2
Cytc	gene encoding cytochrome c
DRP1	dynamin 1-like;
Drp1	gene encoding dynamin 1-like
FIS1	fission mitochondrial 1
Fis1	geneencoding fission mitochondrial 1
IMO	immobilization
MAPK	mitogenactivatedprotein kinase
Mapk1	gene encoding mitogen-activated protein kinase 1
Mapk3	gene encoding mitogen-activated protein kinase 3
Mapk6	gene encoding mitogen-activated protein kinase 6
Mapk8	gene encoding mitogen-activated protein kinase 8
Mapk9	gene encoding mitogen-activated protein kinase 9
Mapk11	gene encoding mitogen-activated protein kinase 11;
Mapk12	gene encoding mitogen-activated protein kinase 12
Mapk13	gene encoding mitogen-activated protein kinase 13
Mapk14	gene encoding mitogenactivated protein kinase 14
MFN1	mitofusin 1
Mfn1	gene encoding mitofusin 1;
MFN2	mitofusin 2
Mfn2	gene encoding mitofusin 2
mtNd1	gene encoding
NADH dehydrogenase 1	mitochondrial
NRF1	nuclear respiratory factor 1
Nrf1	gene encoding nuclear respiratory factor 1
NRF2	nuclear respiratory factor 2;
Nrf2a	gene encoding nuclear respiratory factor 2
OPA1	mitochondrial dynamin like GTPase
Opa1	gene encoding mitochondrial dynamin like GTPase;
OXPHOS	oxidative phosphorylation
PGC1a	peroxisome proliferator-activated receptor gamma coactivator 1-alpha
PGC1b	peroxisome proliferator-activated receptor gamma coactivator 1-beta
PINK1	PTEN induced kinase 1
Ppara	gene encoding peroxisome proliferator-activated receptor alpha
Ppard	gene encoding peroxisome proliferator-activated receptor delta
Ppargc1a	gene encoding peroxisome proliferator-activated receptor gamma coactivator 1-alpha;
Ppargc1b	transcripts for gene encoding peroxisome proliferator-activated receptor gamma coactivator 1-beta
PRKA	protein kinase AMP-activated;
PRKAc	protein kinase AMP-activated catalytic subunit
Prkaca	gene encoding protein kinase cAMP-activated catalytic subunit alpha
Prkacb	gene encoding protein kinase cAMP-activated catalytic subunit beta
Prkar1a	gene encoding protein kinase cAMP-dependent type I regulatory subunit alpha
PRKAR2A	protein kinase cAMP-dependent type II regulatory subunit alpha
Prkar2a	gene encoding protein kinase cAMP-dependent type II regulatory subunit alpha;
Prkar2b	gene encoding protein kinase cAMP-dependent type II regulatory subunit beta
T+DHT	testosterone + dihydrotestosterone
TFAM	transcription factor A mitochondrial
Tfam	gene encoding transcription factor A mitochondrial
UCPs	uncoupling proteins
Ucp1	gene encoding uncoupling protein 1
Ucp2	gene encoding uncoupling protein 2
Ucp3	gene encoding uncoupling protein 3
