# Supplementary material for: Spermatozoa Develop Molecular Machinery to Recover From Acute Stress
Source: Front Endocrinol (Lausanne). 2022 Jul 14;13:896193. doi: 10.3389/fendo.2022.896193 (PMC9329690; doi:10.3389/fendo.2022.896193)
Supplement: Supplementary file 1 [file DataSheet_1.pdf]

Supplementary Material

Supplementary Material file consist of two sections: the supplementary results and supplementary material and methods.

Supplementary results section consists of the presentation of the results obtained from transcriptional analyses and already presented in the main manuscript file, but here the calculation was done using the control form each time point as calibrator (Supplementary Figures 1-7). These results are also presented on the heat maps (Supplementary Figure 8). Also, supplementary results section contains the results of the protein expression analysis in the supernatant samples after the immunoprecipitation analysis of the MFN2 protein (Supplementary Figure 9). Besides, here we presented the results obtained in the *ex vivo* experiments in which spermatozoa from undisturbed control were stimulated with adrenaline for one hour and the mitochondrial membrane potential was measured (Supplementary Figure 10). Lastly, here we show the tables containing the variables loadings from principal component analysis used to graph Figure 11 (Supplementary Tables S1-S3).

Supplementary Material and Methods section contains the key resource table (Supplementary Table S4), the tables related to the primer characteristics and Ct values (Supplementary Tables S5-S11), relative quantification of protein expression after immunoprecipitation analysis as well as the information about antibodies used in the research (Supplementary Table S12**).**

Supplementary Results

Relative expression of transcripts of mitochondrial biogenesis and functionality markers as well as markers of signaling pathways regulating mitochondrial dynamic and spermatozoa functionality calculated using the control group of the same time point as a calibrator.

Calculation of relative gene expression, using the control group of the same time point as a calibrator, showed the changes within each single time point. Transcriptional changes of mitochondrial dynamics and functionality markers in spermatozoa of acutely stressed adult rats are the most prominent at the ZT23 time point, compared to the Control of the same time point. All the followed mitochondrial biogenesis markers (except *Ppara*) and markers of mitochondrial fusion and architecture are significantly increased in spermatozoa from ZT23-1x3hIMO group compared to ZT23-Control. In addition, *Prkn, Cox4i1, Cytc* and *Ucp2* also increased, while *Ucp1* and *Ucp3* decreased, indicating significant transcriptional changes of mitochondrial autophagy and function at the ZT23 time point (Supplementary Figure S1, S2, S3, S4, S5). Also, transcription of the signalling molecules of cAMP and MAPK pathways (regulating mitochondrial dynamics and functionality as well as spermatozoa number and functionality) is the most significantly changed in the spermatozoa of acutely stressed adult rats at ZT23, compared to the Control at the same time point (Supplementary Figure S6, S7).

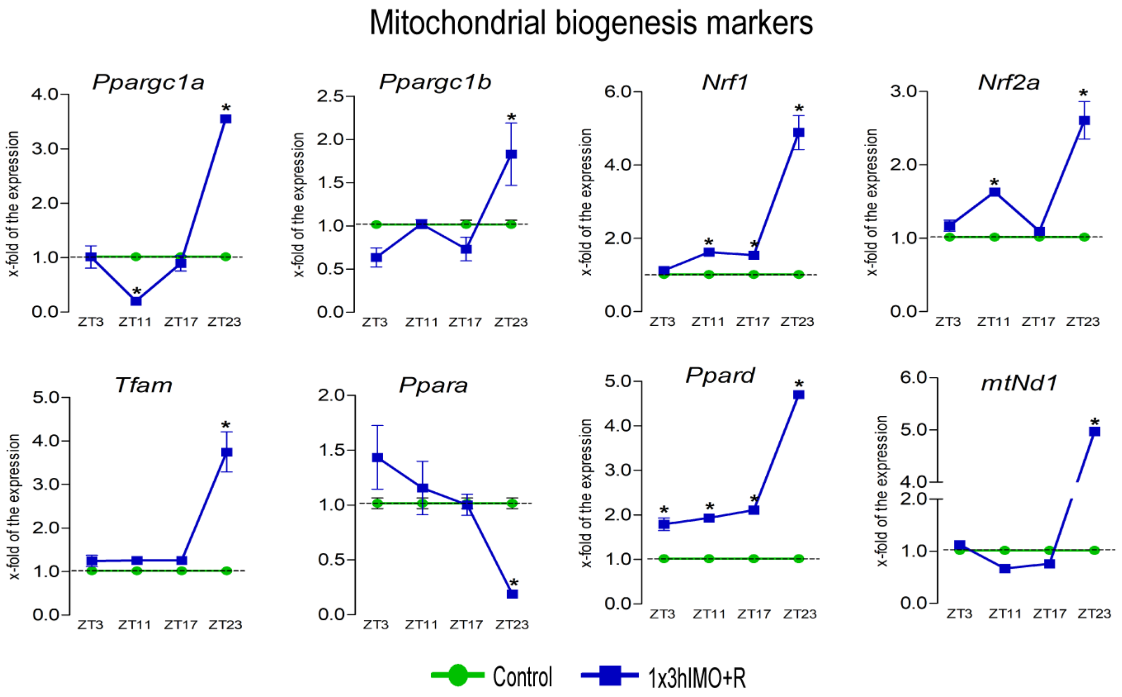

Supplementary Figure S1. Transcription of mitochondrial biogenesis markers in spermatozoa of acutely stressed adult rats is the most prominently changed at the ZT23 time point.

Isolated RNA from spermatozoa of undisturbed and stressed rats was used for analysis of the transcriptional profile of markers of mitochondrial biogenesis. To analyze the effect of acute stress with the recovery period, animals were subjected to immobilization stress (IMO) for 3 hours, once, from ZT0 to ZT3 (1x3hIMO), and allowed to recover (1x3hIMO+R) for 0, 8, 14 and 20 hours after the IMO (ZT3, ZT11, ZT17, and ZT23; ZT0 is a time when the light turned on). In short, the rats were bound in a supine position to a wooden board by fixing the rats' limbs using thread, while the head motion was not limited. Unstressed, freely moving rats were present as a control group (Control) in each experiment. All the activities during the dark phase were performed under the red light. At the end of the experimental period control and stressed animals were quickly decapitated without anesthesia and trunk blood was collected. Individual serum samples were stored at -70 °C until they were assayed for androgens (testosterone + dihydrotestosterone; T+DHT) and corticosterone (CORT) levels. In each experiment, the control and stressed animals randomly divided into four time points groups, with up to 6 animals per time point. The sample size was checked by Power Analysis using G Power software (http://core.ecu.edu/psyc/wuenschk/Power.htm) according to previous results obtained by our group. The experiment was repeated two times.

Data bars are mean ± SEM values of two independent *in vivo* experiments. Statistical significance was set at level $p < 0.05$: * vs. control group of the same time point.

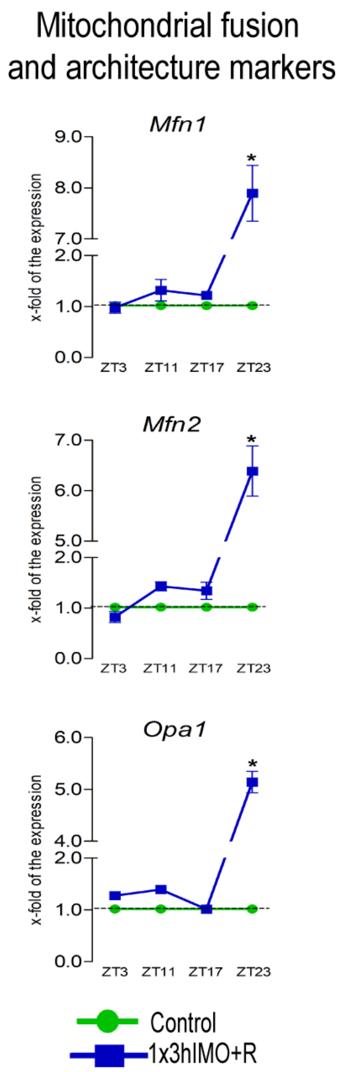

Supplementary Figure S2. Transcription of mitochondrial fusion and architecture markers in spermatozoa of acutely stressed adult rats is the most prominently changed at the ZT23 time point.
Isolated RNA from spermatozoa of undisturbed and stressed rats was used for analysis of the transcriptional profile of markers of mitochondrial fusion and architecture. Data bars are mean ± SEM values of two independent *in vivo* experiments. Statistical significance was set at level $p < 0.05$: * vs. control group of the same time point.

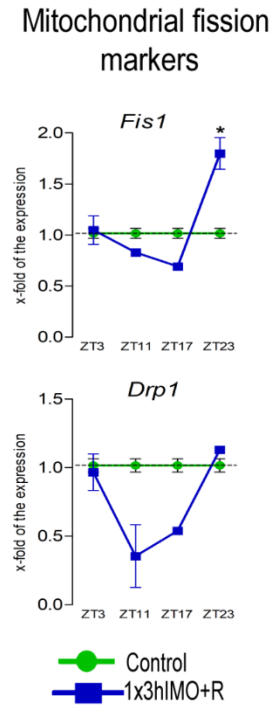

Supplementary Figure S3. Transcription of mitochondrial fission markers in spermatozoa of acutely stressed adult rats is the most prominently changed at the ZT23 time point.

Isolated RNA from spermatozoa of undisturbed and stressed rats was used for analysis of the transcriptional profile of markers of mitochondrial fission. Data bars are mean ± SEM values of two independent *in vivo* experiments. Statistical significance was set at level $p < 0.05$: * vs. control group of the same time point.

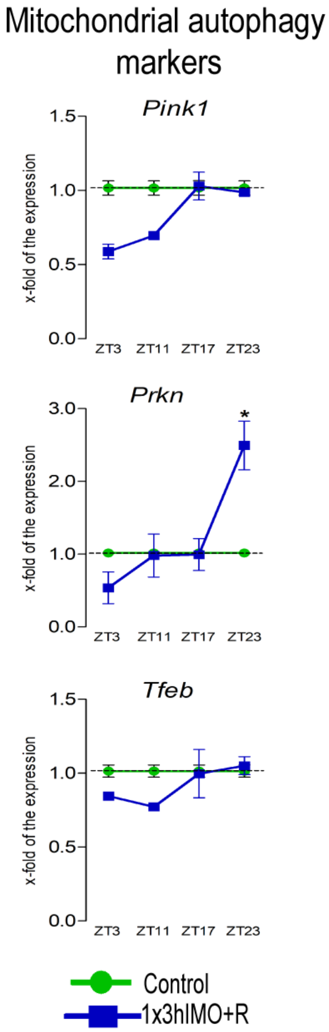

Supplementary Figure S4. Transcription of mitochondrial autophagy markers in spermatozoa of acutely stressed adult rats is the most prominently changed at the ZT23 time point.

Isolated RNA from spermatozoa of undisturbed and stressed rats was used for analysis of the transcriptional profile of markers of mitochondrial autophagy. Data bars are mean ± SEM values of two independent *in vivo* experiments. Statistical significance was set at level $p < 0.05$: * vs. control group of the same time point.

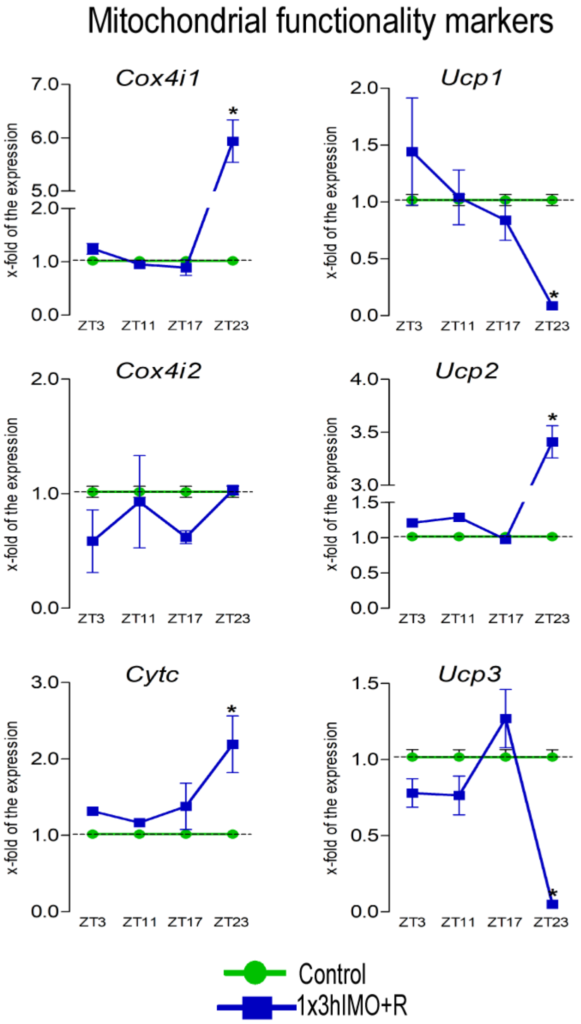

Supplementary Figure S5. Transcription of mitochondrial functionality markers in spermatozoa of acutely stressed adult rats is the most prominently changed at the ZT23 time point.

Isolated RNA from spermatozoa of undisturbed and stressed rats was used for analysis of the transcriptional profile of markers of mitochondrial functionality. Data bars are mean ± SEM values of two independent *in vivo* experiments. Statistical significance was set at level p <0.05: * vs. control group of the same time point.

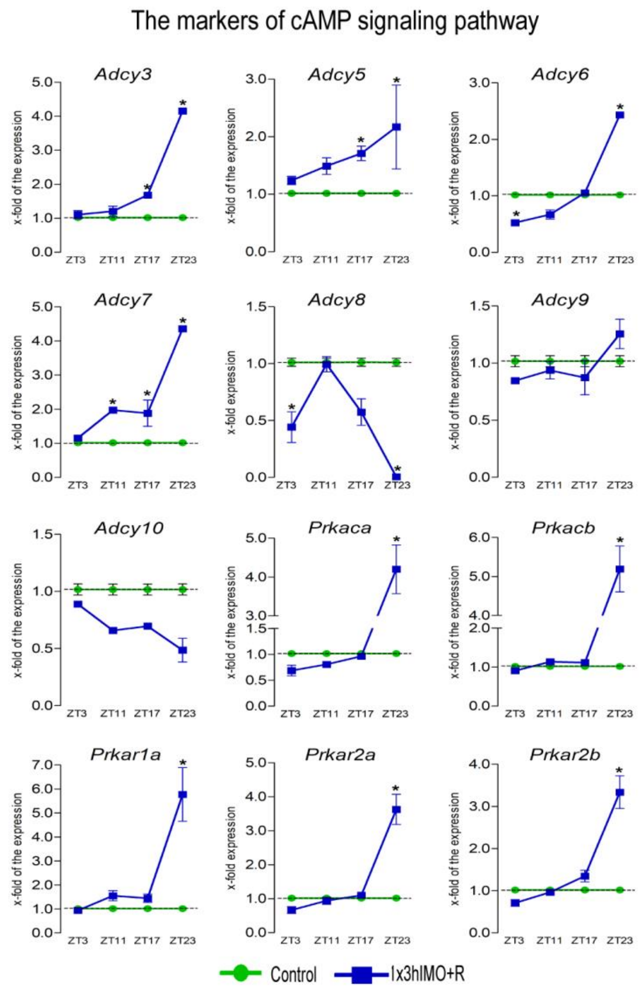

Supplementary Figure S6. Transcription of cAMP signaling markers regulating mitochondrial dynamics and functionality as well as spermatozoa number and functionality in spermatozoa of acutely stressed adult rats is the most prominently changed at the ZT23 time point.

Isolated RNA from spermatozoa of undisturbed and stressed rats was used for analysis of the transcriptional profile of markers of cAMP signaling pathway.

Data bars are mean ± SEM values of two independent *in vivo* experiments. Statistical significance was set at level p <0.05: * vs. control group of the same time point.

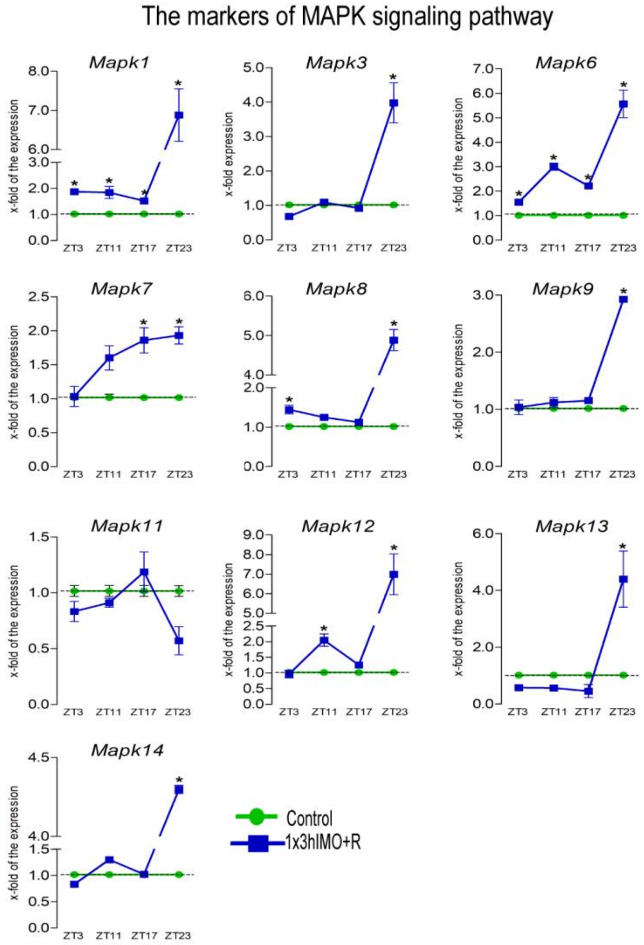

Supplementary Figure S7. Transcription of MAPK signaling markers in spermatozoa of acutely stressed adult rats is the most prominently changed at the ZT23 time point.

Isolated RNA from spermatozoa of undisturbed and stressed rats was used for analysis of the transcriptional profile of markers of MAPK signaling pathway. Data bars are mean ± SEM values of two independent *in vivo* experiments. Statistical significance was set at level $p < 0.05$: * vs. control group of the same time point.

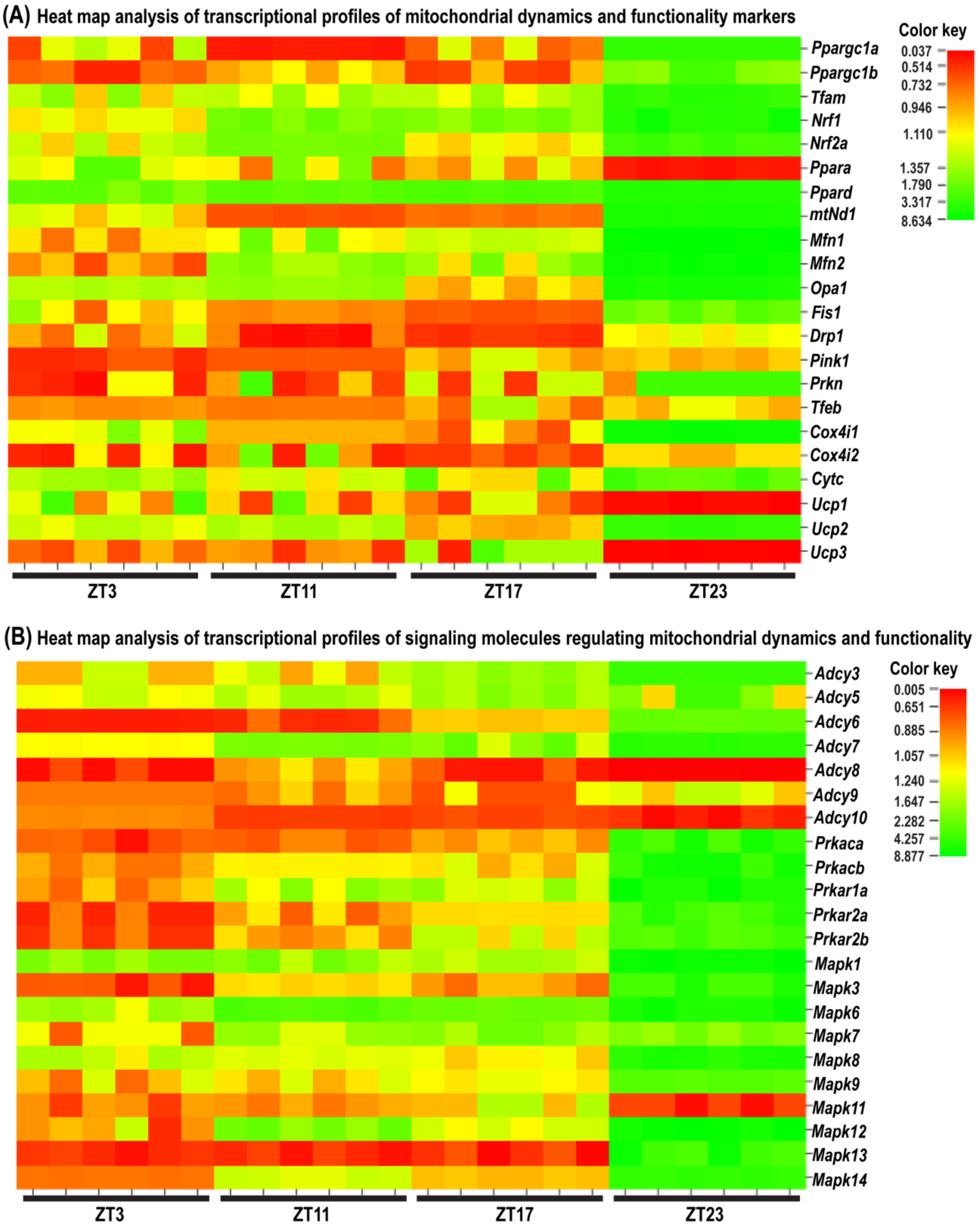

Supplementary Figure S8. Heat map analysis of transcriptional profile of mitochondrial dynamic and functionality markers (A) and signaling molecules regulating mitochondrial dynamics and functionality (B) in spermatozoa of acutely stressed adult rats with recovery at different time points (0hours-ZT3, 8hours-ZT11, 14hours-ZT17, 20hours-ZT23).

The relative fold changes in the genes expression for the before mentioned genes were calculated using the corresponding control group of the same time point as a calibrator to show the changes within each single time point (ZT3, ZT11, ZT17 and ZT23). Color from red to green indicates low to high expression.**Relative protein expression of mitochondrial biogenesis and functionality**

markers as well as markers of signaling pathways regulating mitochondrial dynamic and spermatozoa functionality in spermatozoa samples after MFN2 immunoprecipitation analysis

Considering the relationship of mitofusin 2 expression and motility and cryoprotective potential of human spermatozoa (1), immunoprecipitation analysis of MFN2 protein was performed in spermatozoa samples of stressed animals with different recovery periods. Sprermatozoa samples for immunoprecipitation analysis were lysed and concentration of proteins in each sample was estimated by Bradford method (set at concentration of 300 µg/ml). Pre-clearing of the lysate was done using normal goat serum (Santa Cruz Biotechnology, normal goat serum: sc-2043, (*https://www.scbt.com*)). After pre-clearing step, lysates were mixed with MFN2 antibody (Santa Cruz Biotechnology). Immunoprecipitated complexes with MFN2 antibody were recovered by protein G agarose bead slurry and supernatant was used for further protein analysis using Western blot method. Results show that there is no significant difference in protein expression of ERRα (Supplemental Figure 9A) and ERK1/2 (Supplemental Figure 9C) proteins in all analyzed groups compared to control group of ZT3 time point. Results show that there is significant decrease in 1x3hIMO+R group of ZT3 time point, and oppositely increase in 1x3hIMO+R group of ZT23 time point in the expression of NRF1 protein in supernatant after the immunoprecipitation of MFN2 protein in spermatozoa (Supplemental figure 9B). Relative expression of p38MAPK protein in supernatant after the immunoprecipitation of MFN2 protein, was decreased in stressed groups with recovery periods of 0, 8, 14 and 20 hours (ZT3, ZT11, ZT17 and ZT23), as well as in control groups of ZT11 and ZT17 time points, compared to control group of ZT3 time point (Supplemental Figure 9D). Since immunoprecipitation analysis show protein interaction between MFN2 and PRKAc, analysis for the PRKAc protein in the supernatant was performed. The results show that there is no PRKAc protein present in the supernatant sample after the immunoprecipitation analysis (Supplemental Figure 9E).

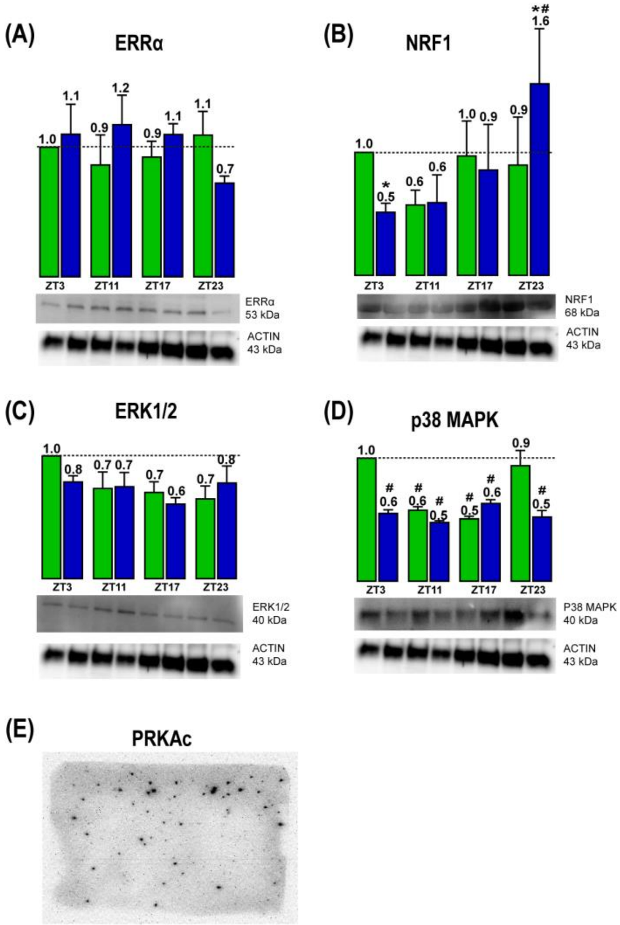

Supplementary Figure S9. Relative expression of ERRα, NRF1, ERK1/2 and p38 MAPK proteins as mitochondrial functionality markers as well as markers of signaling pathways regulating mitochondrial dynamic and spermatozoa functionality in spermatozoa samples after MFN2 immunoprecipitation analysis.

Relative expression of (A) ERRα, (B) NRF1, (C) ERK1/2 and (D) p38 MAPK are analyzed in supernatant after MFN2 immunoprecipitation analysis. Analysis of PRKAc (E) in supernatant after MFN2 immunoprecipitation analysis prove the interaction of the two analysed proteins shown in main manuscript file. Spermatozoa samples for immunoprecipitation analysis were lysed and concentration of proteins in each sample was estimated by Bradford method and set at concentration of 300 µg/ml. Pre-clearing of the lysate was done using normal goat serum. After pre-clearing step, lysates were mixed with MFN2 antibody. Immunoprecipitated complexes with MFN2 antibody were recovered by protein G agarose bead slurry and supernatant was used for further protein analysis using Western blot method. Data bars are mean ± SEM values of two independent *in vivo* experiments. Statistical significance was set at level $p < 0.05$: # vs. control group of the ZT3 time point, * vs. control group of the same time point.

Mitochondrial membrane potential (Δψ) measurement using TMRE fluorescent dye in adult capacitated and non-capacitated spermatozoa after one hour *ex vivo* adrenaline treatment

Three-months-old male Wistar rats were used for the experiment. Adult animals were not subject to any previous treatment. Spermatozoa were isolated from the caudal epididymides and were incubated in capacitated or non-capacitated medium for one hour. After the incubation period, capacitated and non-capacitated spermatozoa were treated *ex vivo* with adrenaline in dose of 10 µM for 3 hours.

To monitor the membrane potential of mitochondria (Δψ) in spermatozoa, tetramethylrhodamine ethyl ester (TMRE) as fluorescent probe was employed according to the procedure published previously (2). Briefly, $1x10^5$ spermatozoa were loaded in each well of a 96-well black plate, with eight replicates from each group. Spermatozoa in a 96-well plate were left for 3 hours in the incubator at 37 °C, to recover from the stimulation procedure. After that period medium was aspirated, TMRE fluorescent dye was added in a final concentration of 100 nM and incubated for 20 minutes at 37 °C. Spermatozoa were washed from TMRE fluorescent dye by aspiration and fluorescence was measured in 0,1% BSA-1xPBS solution (Ex/Em 550/590 nm). After the measurement, all wells were washed with 1xPBS solution and frozen at -20 °C until the measurement of protein concentration in each well by Bradford protein assay.

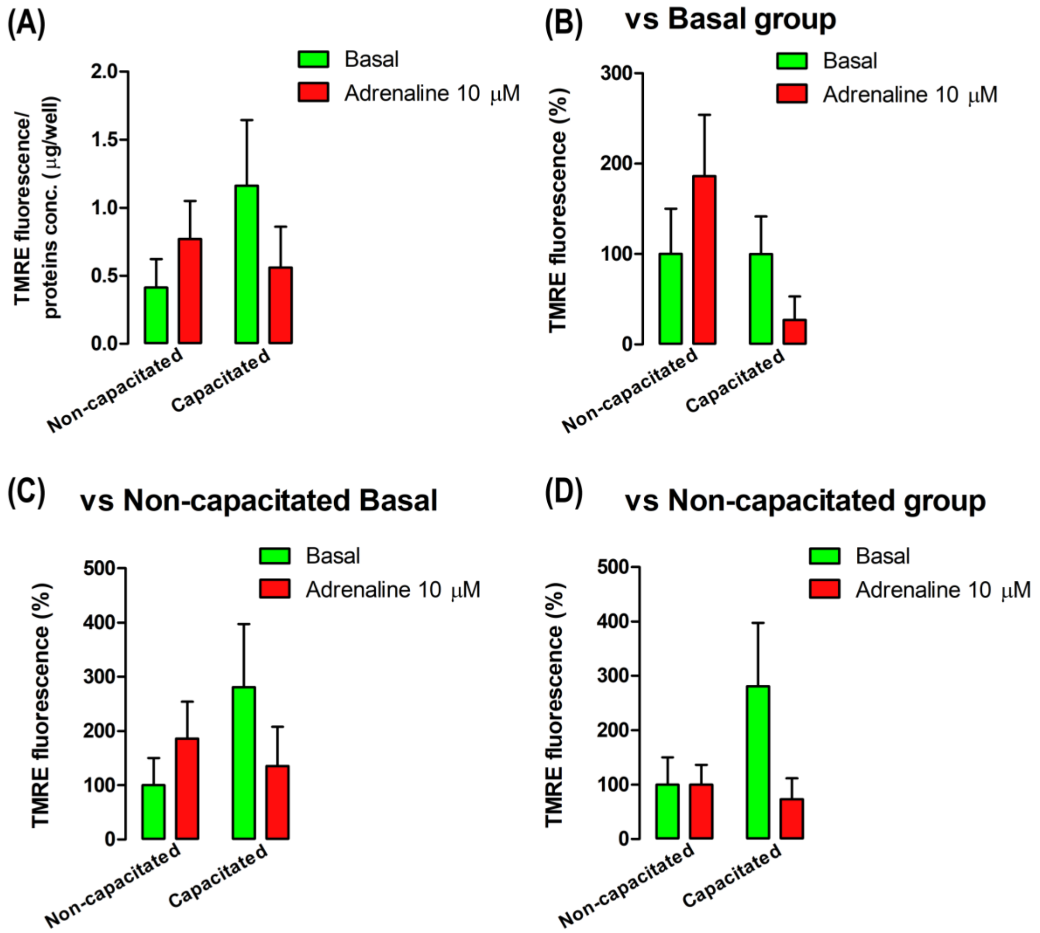

Supplementary Figure S10. The relative ratio of TMRE fluorescence (%) in adult spermatozoa after *ex vivo* adrenaline treatment.

Membrane potential of mitochondria ($\Delta\psi$) in non-treated (basal) and adrenaline treated non-capacitated and capacitated spermatozoa was measured using Tetramethylrhodamine ethyl ester (TMRE) as fluorescent probe. TMRE fluorescence (%) is presented as ratio of TMRE fluorescence and protein concentration is each well.

For each group in the experiment eight replicates was measured. (A) TMRE fluorescence (%) per µg of proteins in each well. (B) TMRE fluorescence (%) calculated on the basal group of non-capacitated and capacitated spermatozoa. (C) TMRE fluorescence (%) calculated on the non-capacitated spermatozoa of basal group. (D) TMRE fluorescence (%) calculated on the non-capacitated group of adrenaline treated and non-treated (basal) spermatozoa.

Data bars are mean ± SEM values of one *ex vivo* experiment.

Supplementary Table S1. Variables loadings from principal component analysis. PC-principal component.

	PC1	PC2	PC3
Ppargc1a	0.250532	-0.15211	-0.12788
Ppargc1b	0.225101	0.202388	0.072176
Tfam	0.254698	0.013513	0.027626
Nrf1	0.254442	0.050704	0.017779
Nrf2a	0.230169	0.235813	-0.05402
Ppara	-0.22415	0.095104	-0.10858
Ppard	0.2604	-0.01874	0.038501
mtNd1	0.2596	-0.00916	-0.08758
Mfn1	0.259605	0.033005	-0.02459
Mfn2	0.260528	0.02916	0.057576
Opa1	0.260379	0.061838	-0.04801
Fis1	0.230016	-0.21855	0.05477
Drp1	0.193326	-0.11477	-0.34203
Pink1	0.008186	0.084998	0.695303
Prkn	0.122627	0.247971	-0.09388
Tfeb	0.229033	-0.00532	0.332673
Cox4i1	0.210243	0.261678	-0.21528
Cox4i2	-0.09302	0.3472	-0.00419
Cytc	-0.01923	0.556862	0.145128
Ucp1	-0.15383	-0.3233	0.050703
Ucp2	0.195433	-0.2827	0.373116
Ucp3	-0.20624	0.2215	0.123077

Supplementary Table S2. Variables loadings from principal component analysis. PC-principal component.

	PC1	PC2	PC3
Adcy3	-0.29879	-0.07554	0.433935
Adcy5	-0.30231	0.142303	0.370199
Adcy6	-0.27565	-0.27288	-0.1054
Adcy7	-0.34729	0.141474	-0.17697
Adcy8	0.15907	-0.59539	0.120152
Adcy9	-0.19952	-0.36001	-0.58736
Adcy10	0.303708	-0.14735	0.309543
Prkaca	-0.32499	-0.04482	-0.19298
Prkacb	-0.33611	-0.05349	0.273228
Prkar1a	-0.10587	-0.59685	0.168749
Prkar2a	-0.3434	-0.01335	0.173834
Prkar2b	-0.34301	0.111726	-0.07994

Supplementary Table S3. Variables loadings from principal component analysis. PC-principal component.

	PC1	PC2	PC3
Mapk1	-0.32048	0.0333	0.451097
Mapk3	-0.37113	0.035601	-0.24431
Mapk6	-0.35524	-0.04676	-0.24312
Mapk7	-0.17178	0.67431	0.265602
Mapk8	-0.28422	-0.4551	-0.1826
Mapk9	-0.35456	0.142737	0.022819
Mapk11	0.206154	0.527175	-0.61479
Mapk12	-0.34248	0.025731	-0.3073
Mapk13	-0.31441	0.186037	0.254423
Mapk14	-0.37242	-0.00804	-0.19021

Supplementary Materials and Methods

Supplementary table S4. Key resources table.

Resource or reagent	Source	Identifier
**Experimental model and biological samples**		
Wistar **rat**	LaRES and ChronAge Laboratories (DBE, Faculty of Sciences, University of Novi Sad, Serbia)	*http://wwwold.dbe.pmf.uns.ac.rs/en/nauka-eng/lares*
**Primary culture of spermatozoa**	Three months-old male rats	NA
**Commercial Reagents/Assays**		
**GenElute Mammalian Total RNA Miniprep Kit**	Sigma Aldrich, Germany	*https://www.sigmaaldrich.com*
**DNase I (RNase-free) treatment**	New England Biolabs, USA	*https://international.neb.com*
**High Capacity Kit for cDNA**	Applied Biosystems/Thermo Fisher Scientific, USA	*https://www.thermofisher.com*
**Power SYBR Green PCR Master Mix**	Applied Biosystems/Thermo Fisher Scientific, USA	*https://www.thermofisher.com*
**Primers**		
**Supplementary tables S5 to S11**	This paper	*www.ncbi.nlm.nih.gov/sites/entrez*
**Antibodies**		
**Rabbit polyclonal anti-PGC-1 (H-300) antibody**	Santa Cruz Biotechnology Inc.	Cat # sc-13067
**Rabbit polyclonal anti-NRF1 (H-300) antibody**	Santa Cruz Biotechnology Inc.	Cat # sc-33771
**Rabbit polyclonal anti-NRF2 (C20) antibody**	Santa Cruz Biotechnology Inc.	Cat # sc-722
**Monoclonal anti-MFN2 (F-5) antibody**	Santa Cruz Biotechnology Inc.	Cat # sc-515647
**Rabbit polyclonal anti-GAPDH (FL-335) antibody**	Santa Cruz Biotechnology Inc.	Cat # sc-25778
**Rabbit polyclonal anti-ERRα (H-80) antibody**	Santa Cruz Biotechnology Inc.	Cat # sc-66882
**Mouse monoclonal anti-PRKAc antibody**	Transduction LaboratoriesTM	Cat # 610981
**Rabbit polyclonal ERK1/2 p44/42 MAPK (ERK1/2)**	Cell Signaling Technolog	Cat #9102
**Rabbit polyclonal p38 MAPK**	Cell Signaling Technology	Cat #9212
**Goat polyclonal ACTIN (I-19)**	Santa Cruz Biotechnology Inc.	Cat # sc-1616
**Software**		

**GraphPad Prism 5 Software**	GraphPad Prism	https://www.graphpad.com/scientific-software/prism

Supplementary table S5. Primers sequences used for the real-time PCR analysis of molecular markers of mitochondrial biogenesis.

Gene	Accession code	Primers	Primer length	Product length	AV Ct
Ppargc1a	NM_031347	F: 5'-AGCCGTAGGCCCAGGTATGACA-3'<br>R: 5'-TGCTTGGCCCTTTCAGACTCCC-3'	22 bp<br>22 bp	107 bp	30.88
Ppargc1b	NM_176075	F: 5'-ACCTTCCGGTGTTCGGAGCATG-3'<br>R: 5'-GTGGAAGGAGGGCTCATTGCGT-3'	22 bp<br>22 bp	81 bp	28.75
Tfam	NM_031326	F: 5'-TATAGTCGTCGGCCCGAGGGAT-3'<br>R: 5'-AAGGCTGACAGGCGAGGGTATG-3'	22 bp<br>22 bp	125 bp	27.92
Nrf1	NM_001100708	F: 5'-GACCATCCAGACGACGCAAGCA-3'<br>R: 5'-ATGGGCGGCAGCTTCACTGTT-3'	22 bp<br>21 bp	136 bp	27.60
Nrf2a	NM_001108841	F: 5'-AGCGGAACTGAACCGCTTGGT-3'<br>R: 5'-GTGACTGGCTGAGCAATCCCGT-3'	21 bp<br>22 bp	84 bp	27.06
Ppara	NM_013196	F: 5'-GTCCTGGAACTGAAGCGACGCT-3'<br>R: 5'-TTACGCCCAAATGCACCACGC-3'	22 bp<br>21 bp	110 bp	28.78
Ppard	NM_013141	F: 5'-ACGGTAAAGGCGGTCCATCTGC-3'<br>R: 5'-TCCTCCTGTGGCTGTTCCATGAC-3'	22 bp<br>23 bp	109 bp	26.49
mtNd1		F: 5' GCGTGGGAGGAGCATCAGGG 3'<br>R: 5' GCGAATGGTCCTGCGGCGT A 3'	20 bp<br>20 bp	271 bp	18.14
Gapdh	NM_017008	F: 5'-TGCCAAGTATGATGACATCAAGAAG-3'<br>R: 5'-AGCCCAGGATGCCCTTTAGT-3'	25 bp<br>20 bp	110 bp	21.08

Primers were designed by using software Primer Express 3.0 (Applied Biosystems) and full genes sequences from the NCBI Entrez Nucleotide database (www.ncbi.nlm.nih.gov/sites/entrez). F - forward; R - reverse.

Supplementary table S6. Primers sequences used for the real-time PCR analysis of molecular markers of mitochondrial fusion and architecture.

Gene	Accession code	Primers	Primer length	Product length	AV Ct
Mfn1	NM_138976.1	F: 5'-CCTTGTACATCGATTCCTGGGTTC-3'<br>R: 5'-CCTGGGCTGCATTATCTGGTG-3'	24 bp<br>21 bp	143 bp	28.63
Mfn2	NM_130894.4	F: 5'-TCAAGCGCCAGTTTGTGGAG-3'<br>R: 5'-CACAGATGAGCAAATGTCCCAGA-3'	20 bp<br>23 bp	118 bp	27.35

Opa1	NM_133585.3	F: 5'-AAAAGCCCTTCCCAGTTCAGA-3'<br>R: 5'-TACCCGCAGTGAAGAAATCCTT-3'	21 bp<br>22 bp	101 bp	26.14
Gapdh	NM_017008	F: 5'-TGCCAAGTATGATGACATCAAGAAG-3'<br>R: 5'-AGCCCAGGATGCCCTTTAGT-3'	25 bp<br>20 bp	110 bp	21.08

Primers were designed by using software Primer Express 3.0 (Applied Biosystems) and full genes sequences from NCBI Entrez Nucleotide database (*www.ncbi.nlm.nih.gov/sites/entrez*). F - forward; R - reverse.

Supplementary table S7. Primers sequences used for the real-time PCR analysis of molecular markers of mitochondrial fission.

Gene	Accession code	Primers	Primer length	Product length	AV Ct
Fis1	NM_001105919.1	F: 5'-ACGCCTGCCGTTACTTCTTC-3'<br>R: 5'-GCAACCCTGCAATCCTTCAC-3'	20 bp<br>20 bp	108 bp	29.75
Drp1	NM_053655.3	F: 5'-AGGTTGCCCGTGACAAATGA-3'<br>R: 5'-CACAGGCATCAGCAAAGTCG-3'	20 bp<br>20 bp	94 bp	27.81
Gapdh	NM_017008	F: 5'-TGCCAAGTATGATGACATCAAGAAG-3'<br>R: 5'-AGCCCAGGATGCCCTTTAGT-3'	25 bp<br>20 bp	110 bp	22.04

Primers were designed by using software Primer Express 3.0 (Applied Biosystems) and full genes sequences from NCBI Entrez Nucleotide database (*www.ncbi.nlm.nih.gov/sites/entrez*). F - forward; R - reverse.

Supplementary table S8. Primers sequences used for the real-time PCR analysis of molecular markers of mitochondrial autophagy.

Gene	Accession code	Primers	Primer length	Product length	AV Ct
Pink1	NM_001106694.1	F: 5'-CAAGCAAGTGTCTGACCCAC-3'<br>R: 5'-GCTTCATACACAGCGGCATT-3'	20 bp<br>20 bp	111 bp	25.69
Prkn	NM_020093.1	F: 5'-CTTCCAGCTCAAGGAAGTGG-3'<br>R: 5'-CAGAGGCATTTGTTTCGTGA-3'	20 bp<br>20 bp	182 bp	32.34
Tfeb	NM_001025707.1	F: 5'-CGACAACATTATGCGCCTGG-3'<br>R: 5'-CTGTACACGTTCAGGTGGCT-3'	20 bp<br>20 bp	102 bp	29.18
Gapdh	NM_017008	F: 5'-TGCCAAGTATGATGACATCAAGAAG-3'<br>R: 5'-AGCCCAGGATGCCCTTTAGT-3'	25 bp<br>20 bp	110 bp	22.04

Primers were designed by using software Primer Express 3.0 (Applied Biosystems) and full genes sequences from NCBI Entrez Nucleotide database (*www.ncbi.nlm.nih.gov/sites/entrez*). F - forward; R - reverse.

Supplementary table S9. Primers sequences used for the real-time PCR analysis of molecular markers of mitochondrial functionality.

Gene	Accession code	Primers	Primer length	Product length	AV Ct
Cox4i1	NM_017202	F: 5'-CGCTGAGATGAACAAGGGCACC-3'<br>R: 5'-TCCCAGATCAGCACAAGCGCA-3'	22 bp<br>21 bp	93 bp	21.88
Cox4i2	NM_053472	F: 5'-CACAGCCCAGGAAGTGCTGCTA-3'<br>R: 5'-TGTGCAGTAAGGCTCATCCGGC-3'	22 bp<br>22 bp	105 bp	32.19
Cytc	NM_012839	F:5'-GCAAGCATAAGACTGGACCAAA-3'<br>R:5'-TTGTTGGCATCTGTGTAAGAGAATC-3'	22 bp<br>25 bp	88 bp	23.56
Ucp1	NM_012682	F: 5'-TCAGCTCTTGTCGCCGGGTTT-3'<br>R: 5'-TGCACAGCTGGGTACACTTGGG-3'	21 bp<br>22 bp	114 bp	29.96
Ucp2	NM_019354	F: 5'-ACGACCTCCCTTGCCACTTCAC-3'<br>R: 5'-GGTACTGGCCCAAGGCAGAGTT-3'	22 bp<br>22 bp	117 bp	22.07
Ucp3	NM_013167	F: 5'-TGCTCAACCCACGGATGTGGT-3'<br>R: 5'-CCTGGCGATGGTTCTGTAGGCA-3'	21 bp<br>22 bp	112 bp	29.81
Gapdh	NM_017008	F: 5'-TGCCAAGTATGATGACATCAAGAAG-3'<br>R: 5'-AGCCCAGGATGCCCTTTAGT-3'	25 bp<br>20 bp	110 bp	21.13

Primers were designed by using software Primer Express 3.0 (Applied Biosystems) and full genes sequences from the NCBI Entrez Nucleotide database (*www.ncbi.nlm.nih.gov/sites/entrez*). F - forward; R - reverse.

Supplementary table S10. Primers sequences used for the real-time PCR analysis of cAMP signaling elements.

Gene	Accession code	Primers	Primer length	Product length	AV Ct
Adcy3	NM_130779	F: 5'-GCATCGAAACCTACCTCATCA-3'<br>R: 5'-TGGGCTCCTTGGTCTCA ATAA-3'	21 bp<br>21 bp	141 bp	31.76
Adcy5	NM_022600	F: 5'-AACCAGGTGAACGCATGTCA-3'<br>R: 5'-CTCTGGGAAGTTGCAGTTGGA-3'	20 bp<br>21 bp	105 bp	29.95
Adcy6	NM_012821	F: 5'-CTGCCTCAGCCTGCTTATGTG-3'<br>R: 5'-GGAGTCCTGGCGGAAGCT-3'	21 bp<br>18 bp	99 bp	26.86
Adcy7	NM_053396	F: 5'-TTCCGTGCGTGTAACCCGCT-3'<br>R: 5'-GCCTTCTGCCTCCGTCCGTT-3'	20 bp<br>20 bp	123 bp	26.55
Adcy8	NM_017142	F: 5'-ATTGCCTCAGTGGTGACTA-3'<br>R: 5'-CAAACTCTCCTCGGGCT-3'	19 bp<br>17 bp	113 bp	28.83
Adcy9	NM_001106980	F: 5'-TCACCAAGCTGTACGCCCGG-3'<br>R: 5'-GGGCTGTCAACACGTCCCGA-3'	20 bp<br>20 bp	124 bp	29.13
Adcy10	NM_021684	F: 5'-CCAGGCATCGTGACCTGCGA-3'<br>R: 5'-ACTGGTCCGGGATCCGCAAC-3'	20 bp<br>20 bp	113 bp	30.64
Prkaca	NM_001100922.1	F: 5'-TCAGTGAGCCCCACGCCCGTT-3'<br>R: 5'-TCTCGGGCTTCAGGTCCCGG-3'	21 bp<br>20 bp	99 bp	25.59
Prkacb	NM_001077645	F: 5'-GGGTCATGGGGAACACGGCG-3'<br>R: 5'-CCAGCATTACTCGGGGGAGGGT-3'	20 bp<br>22 bp	124 bp	26.98
Prkar1a	NM_013181	F: 5'-TGTGCTGCAGCGTCGGTCAG-3'<br>R: 5'-AGTGGCAGCCCGAGGACGAT-3'	20 bp<br>20 bp	112 bp	23.77
Prkar2a	NM_019264	F: 5'-GCCCGACCTCGTCGACTTCG-3'<br>R: 5'-TCCTGCGCGTGAAAGGTCGT-3'	20 bp<br>20 bp	108 bp	26.81
Prkar2b	NM_001030020	F: 5'-CCCATGCGCTCCGATTCCGA-3'<br>R:5'-GCACATACCGAGGCACGCCT-3'	20 bp<br>20 bp	107 bp	28.70
Gapdh	NM_017008	F: 5'-TGCCAAGTATGATGACATCAAGAAG-3'<br>R: 5'-AGCCCAGGATGCCCTTTAGT-3'	25 bp<br>20 bp	110 bp	21.12

Primers were designed by using software Primer Express 3.0 (Applied Biosystems) and full genes sequences from NCBI Entrez Nucleotide database (*www.ncbi.nlm.nih.gov/sites/entrez*). F - forward; R - reverse.

Supplementary table S11. Primers sequences used for the real-time PCR analysis of MAPK signaling elements.

Gene	Accession code	Primers	Primer length	Product length	AV Ct
Mapk1	NM_053842.1	F: 5'-GTTCTGCACCGTGACCTCAAG-3'<br>R: 5'-GCAAGGCCAAAGTCACAGATC-3'	21 bp<br>21 bp	80 bp	26.12
Mapk3	NM_017347.2	F: 5'-TCCCTCTCAAGCTGCCACAT-3'<br>R: 5'-ACATCCAATCACCCACACACA-3'	20 bp<br>21 bp	60 bp	25.86
Mapk6	NM_031622.2	F: 5'-CATTTGAACTGGCATGTCGTTT-3'<br>R: 5'-CCTGCACTGCATTGTTTTGC-3'	22 bp<br>20 bp	62 bp	25.61
Mapk7	NM_001191547.1	F: 5'-GCCCCTTCCACTAGCCTTTT-3'<br>R: 5'-GAACCAGGCAACCCACTAGGT-3'	20 bp<br>21 bp	62 bp	27.40
Mapk8	NM_053829.2	F: 5'-TCAACGTCTGGTATGATCCTTCA-3'<br>R: 5'- CTGCTTGTCAGGGATCTTTGG-3'	23 bp<br>21 bp	62 bp	27.26
Mapk9	NM_017322.1<br>NM_001270544.1<br>NM_001270545.1	F: 5'-GGAAGGCTGCCGATGAAA-3'<br>R: 5'-AGCCAGAGTCCTTCACAGACAAG-3'	18 bp<br>23 bp	57 bp	26.97
Mapk11	NM_001109532.2	F: 5'-GGGCGCTGACCTGAATAACA-3'<br>R: 5'-GCAGCAGCTGGTAGACAAGGA-3'	20 bp<br>21 bp	80 bp	29.84
Mapk12	NM_021746.1	F: 5’-GGATGTGTTCACTCCCGATGA-3’<br>R: 5’-CCAGGTCAGTGCCCATGAAT-3’	21 bp<br>20 bp	80 bp	26.23
Mapk13	NM_019231.2	F: 5’-CTGGTCTGTTGGCTGCATCA-3’<br>R: 5’-TCAGCTGGTCCAGGTAGTCCTT-3’	20 bp<br>22 bp	80 bp	27.04
Mapk14	NM_031020.2	F: 5’-GCTGTCGACCTGCTGGAAAA-3’<br>R: 5’- TAGGCATGCGCAAGAGCTT-3’	20 bp<br>19 bp	80 bp	25.28
Gapdh	NM_017008	F: 5'-TGCCAAGTATGATGACATCAAGAAG-3'<br>R: 5'-AGCCCAGGATGCCCTTTAGT-3'	25 bp<br>20 bp	110 bp	20.94

Primers were designed by using software Primer Express 3.0 (Applied Biosystems) and full genes sequences from NCBI Entrez Nucleotide database (*www.ncbi.nlm.nih.gov/sites/entrez*). F - forward; R - reverse.

Relative quantification of protein expression after immunoprecipitation analysis

Sprermatozoa samples for immunoprecipitation analysis were lysed in 1 ml buffer containing 20 mM HEPES, 10 mM EDTA, 2.5 mM MgCl2, 40 mM β-glycerophosphate, 1mM DTT, 1% NP-40, 0.5 mM 4-(aminoethyl)-benzenesulfonyl fluoride hydrochloride, 1 µM aprotinin, 2 µM leupeptin and Phosphatase inhibitor cocktail tablets (cont. (1R, 2S, 3R, 6S)-1.2-dimethyl-3.6-epoxycyclohexane-1.2-dicarboxylic anhydride). Concentration of proteins in each sample was estimated by Bradford method and set at concentration of 300 µg/ml. Equal amount of protein in each sample (300 µg) was used for the immunoprecipitation. Pre-clearing of the lysate was done using 5 µl of normal goat serum (Santa Cruz Biotechnology, normal goat serum: sc-2043, (*https://www.scbt.com*)) mixed with 1 ml of lysate and incubated on ice for 1 hour. After the incubation 100 µl of bead slurry was added to each sample and incubated for 30 minutes at 4 °C with gentle agitation. Supernatant for the immunoprecipitation was collected after 10 minutes at 14 000*xg* at 4 °C centrifugation. After pre-clearing process, lysates were mixed with MFN2 antibody (Santa Cruz Biotechnology) and incubated at 4 °C overnight with constant rotation. During additional overnight incubation at 4 °C with constant

rotation, immunoprecipitated complexes with MFN2 antibody were recovered by 80 µl of protein G agarose bead slurry and supernatant was used for further protein analysis by Western blot. Western blot analysis was done in the same manner as the analysis described in the main manuscript text and as described previously (1). Immune-reactive bands were detected using MyECL Imager (Thermo Fisher Scientific Inc.; *https://www.thermofisher.com*) and analyzed as two-dimensional images using Image J version 1.48 (*http://rsbweb.nih.gov/ij/download.html*). The optical density of images is expressed as volume adjusted for the background, which gives arbitrary units of adjusted volume. Normalization of the data was done using ACTIN protein expression, as the endogenous control. Immune-detection was performed with different antibodies (all details are listed in Supplementary Table S12).

Supplementary table S12. The characteristics of the antibodies.

**Target**	**Name of Antibody**	**Antigen sequence**	**Manufacturer, catalog #**	**Mono- or polyclona**	**Dil. used**
**PGC1**	PGC-1 (H-300): sc-13067	Amino acids 1-300 mapping near the N-terminus of PGC-1 of human origin	Santa Cruz Biotechnology Inc.<br>sc-13067	Rabbit polyclonal antibody	1:500
**NRF1**	NRF-1 (H-300): sc-33771	Amino acids 204-503 mapping near the C-terminus of NRF-1 of human origin	Santa Cruz Biotechnology Inc.<br>sc-33771	Rabbit polyclonal antibody	1:200
**NRF2**	NRF2 (C-20):sc-722	Peptide maping at the C-terminus of Nrf2 of human origin	Santa Cruz Biotechnology Inc.<br>sc-722	Rabbit polyclonal antibody	1:100
**MFN2**	Mfn2 (F-5): sc-515647	Amino acids 461-528 mapping within a cytoplasmic domain of Mfn2 of human origin	Santa Cruz Biotechnology Inc.<br>sc-515647<br>Mw (MFN2) = 57/100 kDa	Mouse monoclonal antibody	8 μg
**ERRα**	ERRα (H-80): sc-66882	Amino acids 81-160 mapping near the N-terminus of ERRα of human origin	Santa Cruz Biotechnology Inc.<br>sc-66882<br>Mw (ERRα) = 53 kDa	Rabbit polyclonal antibody	1:100
**PRKAc**	Purified Mouse Anti-PKA[C]	Amino acids 18-347 of PKA[Cα] of human origin.	BD Transduction Laboratories™	Mouse monoclonal antibody	1:500
**ERK1/2**	p44/42 MAPK (ERK1/2) Antibody #9102	Polyclonal antibodies are produced by immunizing animals with a sintetic peptide (KLH-coupled) derived from sequence in the C-terminus of rat p44 MAP kinase.	Cell Signaling Technology<br>#9102<br>MW (ERK1/2) = 42, 44 kDa	Rabbit polyclonal antibody	1:500
**p38 MAPK**	p38 MAPK Antibody #9212	Polyclonal antibody is produced by immunizing animals with a sintetic peptide coresponding to the sequence of human p38 MAPK. P38 MAPK antibody detects endogenous levels of total p38α, -β or –γ MAPK protein.	Cell Signaling Technology<br>#9212<br>MW (p38 MAPK) = 43 kDa	Rabbit polyclonal antibody	1:500

**ACTIN**	Actin (I-19): sc-1616	Polyclonal affinity purified antibody raised against a peptide mapping at the C-terminus of Actin of human origin.	Santa Cruz Biotechnology Inc.<br>sc-1616<br>Mw (Actin) = 43 kDa	Goat polyclonal antibody	1:100
**GAPDH**	Anti GAPDH (FL-335) antibody	Amino acids 1-335 representing full length GAPDH of human origin	Santa Cruz Biotechnology Inc.<br>sc-25778	Rabbit polyclonal antibody	1:150

References

1. Fang F, Ni K, Shang J, Zhang X, Xiong C, Meng T. Expression of mitofusin 2 in human sperm and its relationship to sperm motility and cryoprotective potentials. *Exp Biol Med* (2018) 243:963–969. doi: 10.1177/1535370218790919

2. Gak IA, Radovic SM, Dukic AR, Janjic MM, Stojkov-Mimic NJ, Kostic TS, Andric SA. Stress triggers mitochondrial biogenesis to preserve steroidogenesis in Leydig cells. *Biochim Biophys Acta BBA - Mol Cell Res* (2015) 1853:2217–2227. doi: 10.1016/j.bbamcr.2015.05.030
